# Microstructure and mechanical properties of friction stir spot welded AA5052-H112 aluminum alloy

**DOI:** 10.1016/j.heliyon.2021.e06009

**Published:** 2021-02-01

**Authors:** Tiwan, M. Noer Ilman, Kusmono

**Affiliations:** a Department of Mechanical and Industrial Engineering, Faculty of Engineering, Universitas Gadjah Mada, Jln. Grafika No. 2 Yogyakarta 55281, Indonesia; b Department of Mechanical Engineering Education, Yogyakarta State University Yogyakarta, Indonesia

**Keywords:** Friction stir spot welding (FSSW), AA5052-H112, Pin geometry, Rotational speed, Microstructure, Mechanical properties

## Abstract

The influences of the pin geometry and rotational speed on the microstructure evolution and mechanical properties of the AA 5052-H112 FSSW joint were investigated. Results showed that hook height and fully bonded region (FBR) width were significantly dependent by both pin geometry and rotational speeds. Both pin geometry and tool rotational speed had an apparent influence on the strength of the weld. At 900 and 1400 rpm, the strength of welds prepared using a cylindrical pin was higher compared to that of a step pin. For the step pin, the cross-tension strength of the welds increased as rotational speed increased. For cylindrical pin, the tensile/shear and cross-tension loads achieved the maximum values of 3589 and 3419 N at 1400 rpm, respectively. Under tensile/shear loading, shear and tensile/shear fractures were observed. On the other hand, under cross-tension loading, two types of fracture modes, namely nugget debonding and nugget pull-out were detected.

## 1 Introduction

Recently, the use of aluminum-based materials in the vehicle industries is increasing. Aluminum, which has lower specific gravity than steel, can meet the demands of creating lightweight vehicles. The use of the lightweight materials in vehicles production became favorable method for power usage and fuel consumption reduction [1, 2]. The 5XXX series Al–Mg aluminum alloy is one type of aluminum alloys that has received great attention in the automotive manufacturing industry because of its excellent strength to weight ratio, corrosion resistance, weldability and recycling potential [3]. The strength of aluminum can be designed to be equivalent to steel through the addition of alloy elements. In terms of manufacturing, aluminum-based materials can be easily formed and machined [4, 5].

AA5052 aluminum alloy has been extensively applied for the manufacture of vehicle components, ships, and vessels. This aluminum can be joined by a fusion welding process such as TIG (Tungsten Inert Gas) [6], MIG (Metal Inert Gas), and RSW (Resistance Spot Welding). It has been well known that the aluminum welding is more difficult than steel because the aluminum characteristics are very different from steel. Some characteristics include such as the existence of aluminum oxide layer on the surface, high thermal conductivity and thermal expansion coefficient, and low melting temperature [7]. Moreover, some welding defects such as porosity, solidification, and liquidation cracking often occur during the welding process in aluminum welds or after the welding [8].

To overcome the lack of fusion welding, friction-based solid-state welding methods such as FSSW (Friction Stir Spot Welding) can be used to solve this problem. FSSW is a friction-based spot welding that can replace the RSW (Resistance Spot Welding) [9]. RSW is difficult to implement on aluminum because aluminum has high electrical conductivity, low electrical resistance, and an aluminum oxide layer on the surface that has a high melting point [7]. The aluminum welding with the FSSW method is done in a solid condition so that defects such as porosity, solidification, and liquidation cracking can be avoided on the weld. This method is very suitable for welding of aluminum materials that are susceptible to melt [9].

The FSSW method is a new development of the FSW (Friction Stir Welding) which was introduced in 1993 [10] and while the FSW was firstly introduced in 1991 [11]. Studies on the impacts of FSSW parameters such as tools and welding parameters on the microstructural evolution and mechanical characteristics of various types of aluminum alloys have been investigated [12]. The tool is the main equipment for welding which creates a welded structure that is typical of FSSW welding. The welding parameters are magnitude factors that influence weld results consisting of rotational speed (RS), plunge rate (PR), plunge depth (PD), and dwell time (DT) when the tool turns when it reaches maximum depth [13, 14, 15].

FSSW has been applied to several types of aluminum alloys such as AA2024 [15], AA5182 [16], AA6061 [17], AA3003 [18], and AA7050 [19]. Other metals that have been successfully joined by the FSSW process are magnesium alloys [20], steel [21], copper [22], and also for composite materials [23]. The tool geometry largely determines the microstructure evolution and mechanical characteristics of the welded joints [24, 25, 26, 27, 28]. The tool geometry influences the pattern of material flow under the tool during the deformation and stirring process on FSSW [25]. As a result of the deformation and stirring processes, a hook is formed which is a characteristic of the FSSW weld structure. Stir zone width and hook dimensions are influenced by the geometry of the tool [26]. Badarinarayan et al. [27] used cylindrical and triangular pins and it was found that the static strength of FSSW AA 5083-O joints using triangular pin was twice compared to that of the cylindrical pin. Badarinarayan et al. [28] studied the influence of pin geometry (cylindrical and triangular pins) on the hook geometry and the static strength of FSSW AA 5754-O. They found that weld joints using a cylindrical pin (FSSW–C) produced continuous hook and an extensive stir zone in the weld zone whereas the joints using a triangular pin (FSSW-T) resulted in a seized hook but a much smaller stir zone. The welds produced by a triangular pin showed higher strength compared to a cylindrical pin. They concluded that the pin geometry also obviously influenced the hook geometry and the SZ shape, especially at the low rotational speed [15].

In addition to pin geometry, the important factors that determine the quality of the FSSW weld are the FSSW parameters consisting of RS, PR, PD, and DT. The shear strength of the FSSW joins increases with increasing RS and reaches its optimum value at 1250 rpm [29]. RS and hook height have a directly proportional relationship, as the RS increases, the hook height increases. PD is the most influencing factor for weld strength and then followed by DT and RS [30]. At low RS, the shear strength will increase with increasing DT. However, the shear strength of the FSSW is more influenced by the RS than the DT [31]. Effective welding width and hook height increase with increasing PR from 2 - 10 mm/min but the effect is not significant at higher PR [32].

FSSW has been extensively used to several aluminum alloys including AA5XXX series and investigation on the impact of welding parameters on the characteristics of FSSW welds have been reported previously. AA 5052 is aluminum alloy with a main alloying element of Mg which has moderate strength. The strength of AA 5052 alloy can be improved by applying strain hardening. A few studies on the influences of welding parameters (rotational speeds and dwell time) on properties of FSSW AA5052-H112 alloy have been reported [2, 14]. However, the effects of pin geometry and rotational speeds on the microstructural evolution and mechanical characteristics of FSSW AA5052-H112 have been limited. Therefore, studies on the impacts of both pin geometry and rotational speed on the microstructural and mechanical characteristics of FSSW in 3 mm thick AA 5052-H112 aluminum alloy were discussed in this work. Two different pin geometry, namely cylindrical and step pins were used in this work and the rotational speed was varied for 900, 1400, and 1800 rpm.

## 2 Experimental procedure

**Figure 1:**
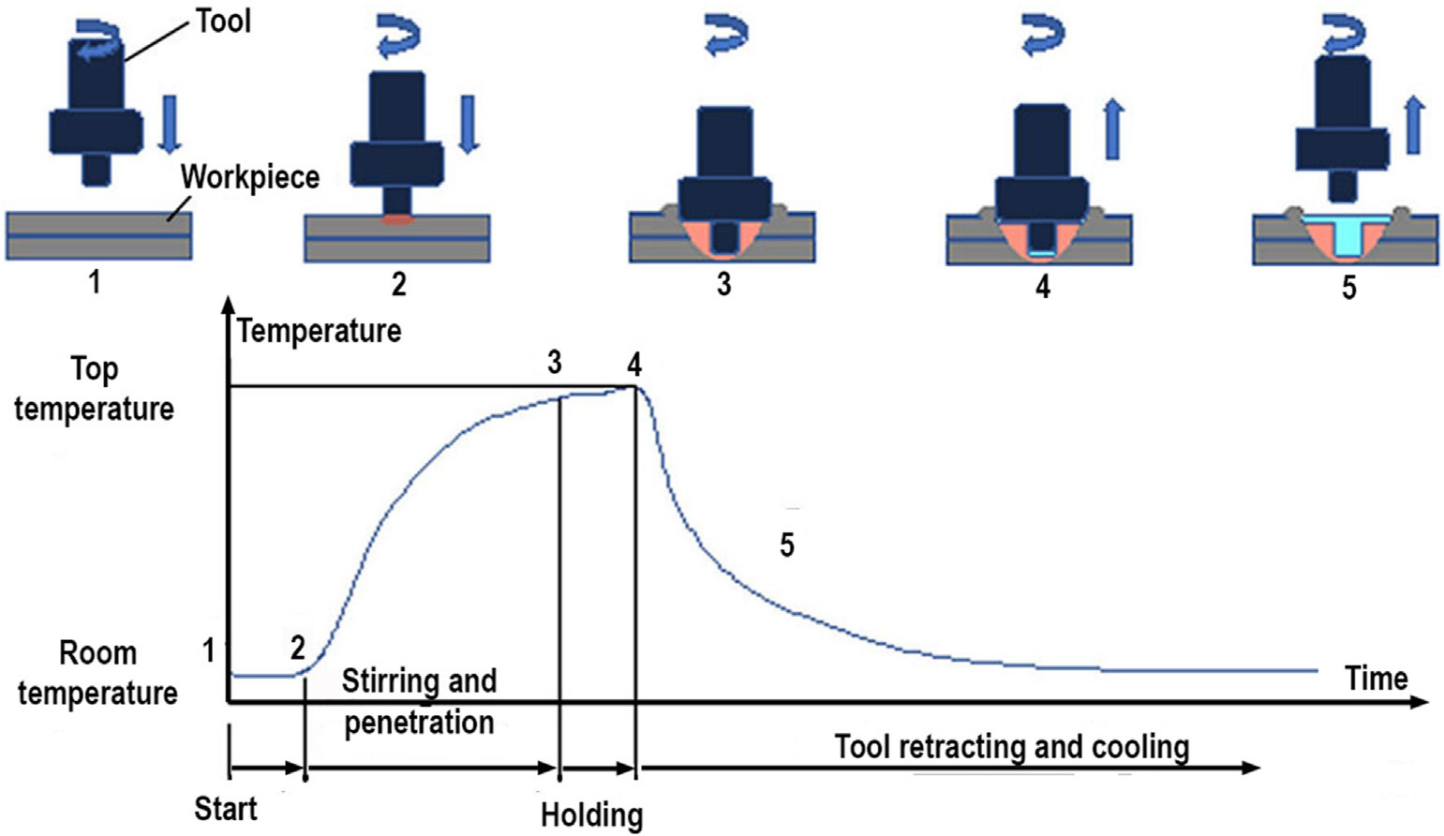
Schematic illustrations of the FSSW process.

The base material used in the FSSW was AA5052-H112 aluminum alloy sheet with 3 mm in thickness with the chemical composition as presented in Table 1. The FSSW tools were made from H13 tool steel which was hardened by the quenching process [33, 34]. The welding process was carried out using the milling machine by adjusting the spindle rotation speed. The spindle could move vertically up and down. The machine table could be moved horizontally lengthwise, transversely, and vertically up and down. Placement and clamping of workpieces use jig which was mounted on the machine table. A schematic overview of the FSSW process can be seen in Figure 1. At the stages of 1–2, the tool rotates and starts moving downwards until it hits the workpiece surface. Initially, the workpiece is still at room temperature and changes occur when the tool touches the workpiece surface. At stages of 2–3, a tool that has touched the workpiece surface, the tool pushes inward to a predetermined depth. The workpiece temperature starts to rise and continues to rapidly increase up to position 3. At the stages of 3–4, the tool remains rotating and is held at a fixed depth of 0.1 mm for a constant dwell time of 5 s. The temperature of the workpiece increases slightly until it reaches its peak (position 3–4). At the stages of 4–5, the tool is retracted from the workpiece, the joint between the upper and the lower sheets is completed in the areas where the plastic flow occurred. Initially, the workpiece temperature drops rapidly and slows down to room temperature during the cooling process (position 4–5).

**Table 1:** Chemical compositions of AA5052-H112 aluminum alloy.

Chemical composition (wt. %)							
Mg	Si	Cu	Fe	Zn	Mn	Cr	Al
2.20–2.80	0.25	0.10	0.40	0.10	0.10	0.15–0.35	Bal.

**Figure 2:**
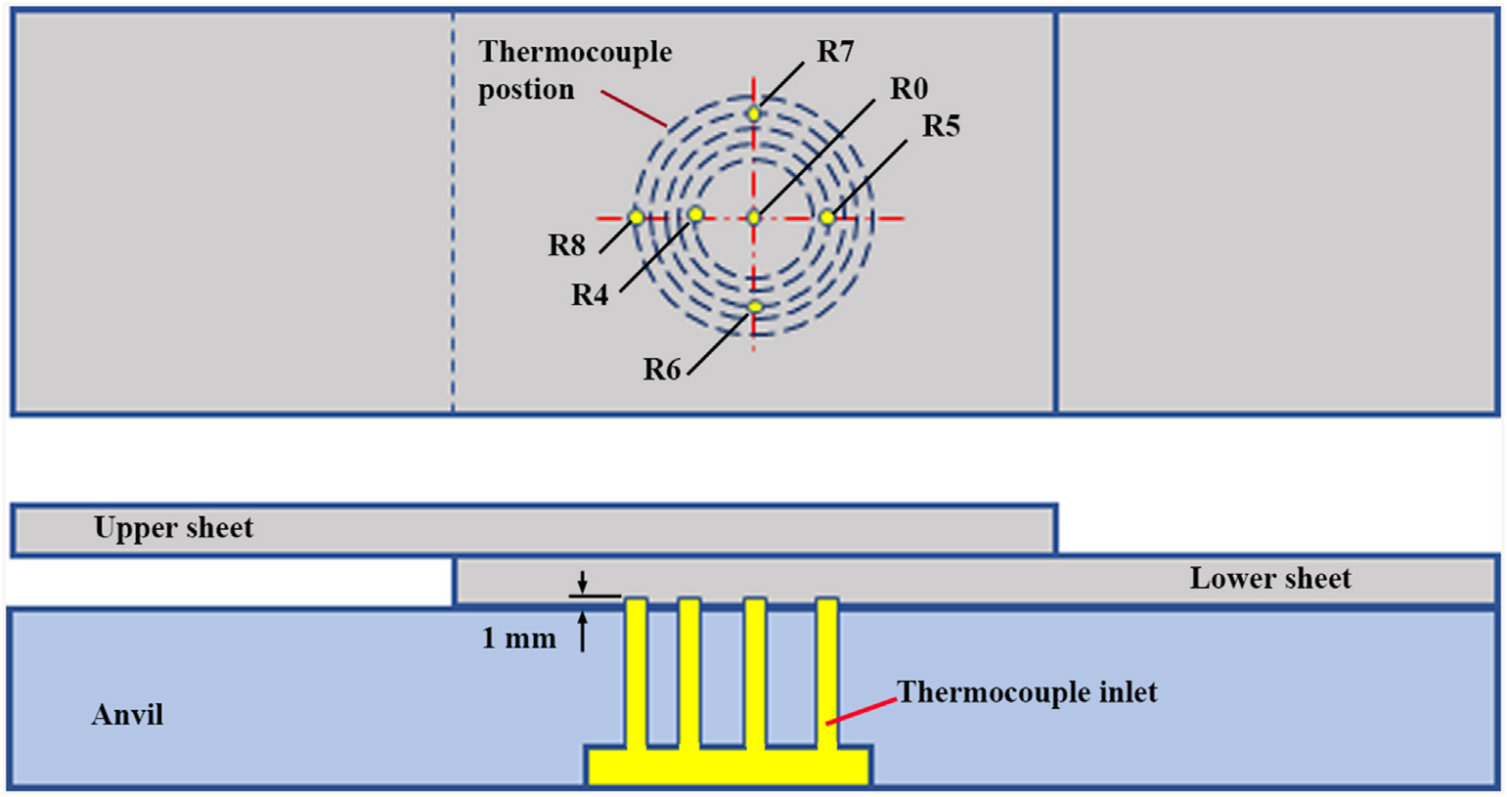
Position and placement of the thermocouple on the FSSW weld.

**Figure 3:**
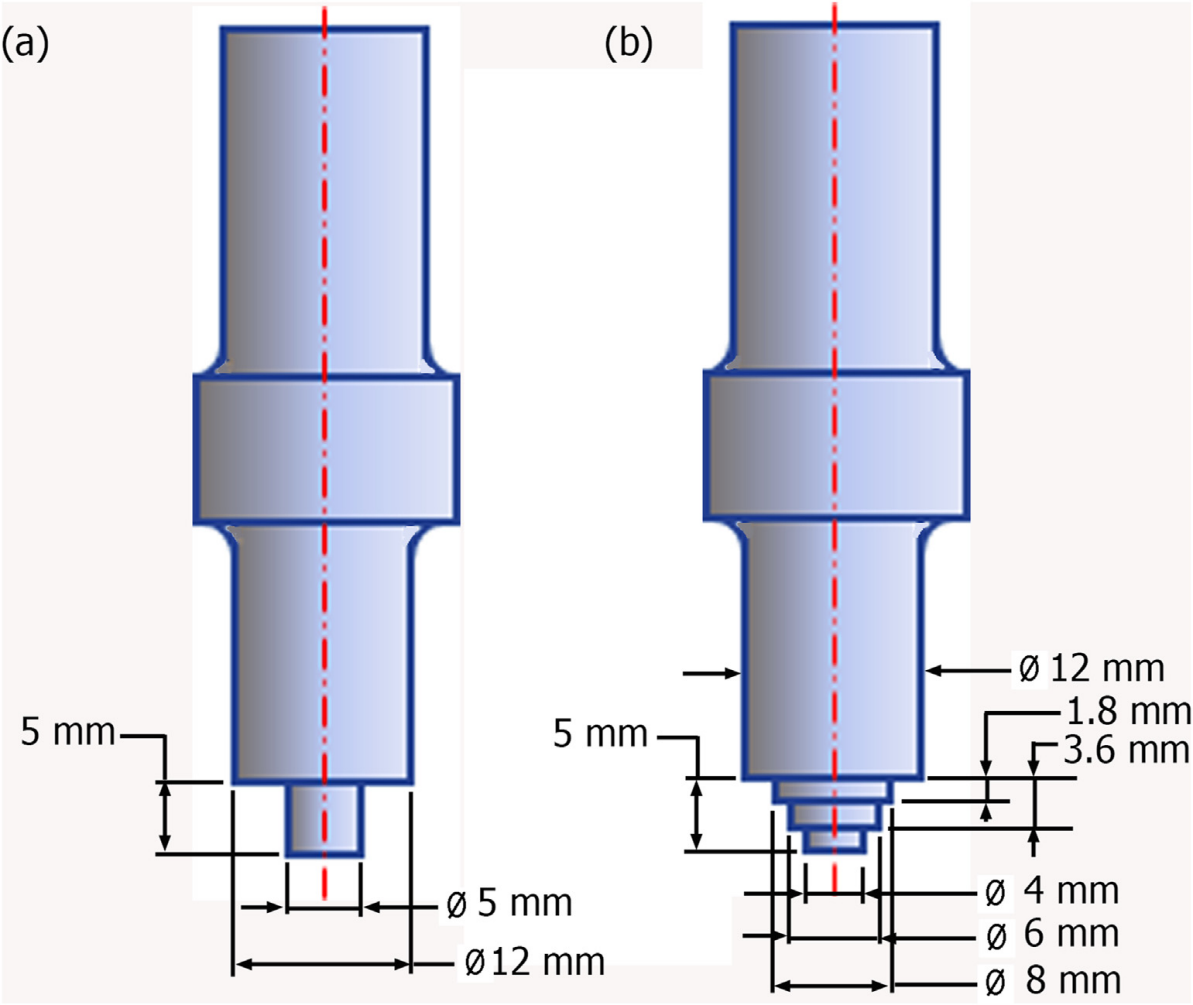
Geometry of pins used: (a). Cylindrical pin (b). Step pin.

Temperature measurements were performed using thermocouples and data loggers. The position and placement of the thermocouple during the FSSW process were exhibited in Figure 2. The tensile/shear and cross-tension specimens were produced following ISO 14273 and ISO 14272 standards, respectively. All specimens were welded in lap configuration. The tensile/shear specimens were prepared by a sheet of 120 mm × 30 mm with an overlap area of 30 mm × 30 mm, and the cross-tension specimens were prepared using two sheets of 100 mm × 30 mm with an overlap area of 30 mm × 30 mm. The FSSW process was carried out with variations of pin geometry and tool rotational speeds. There were two types of pin tools used in this study, namely cylindrical and step pins as presented in Figure 3. The rotational speed (RS) variations of the tool were 900, 1400, and 1800 rpm. The selection of the rotation speed ranges referred to previous findings demonstrated by Bozzi et al. [16] and Padmanaban et al. [35]. Bozzi et al. [16] presented that the tensile/shear strength of FSSW AA5182 aluminum alloy enhanced with increasing the rotational speed up to 1300 rpm but then decreased at above 1300 rpm. In addition, Padmanaban et al. [36] also demonstrated that the tensile/shear strength of the FSSW AA6061 aluminum alloy enhanced by increasing speed up to 1200 rpm but then decreased at speed over 1200 rpm. Other FSSW parameters such as PD of 0.1 mm, PR of 4 mm/min and DT of 5 s were kept constant.

**Figure 4:**
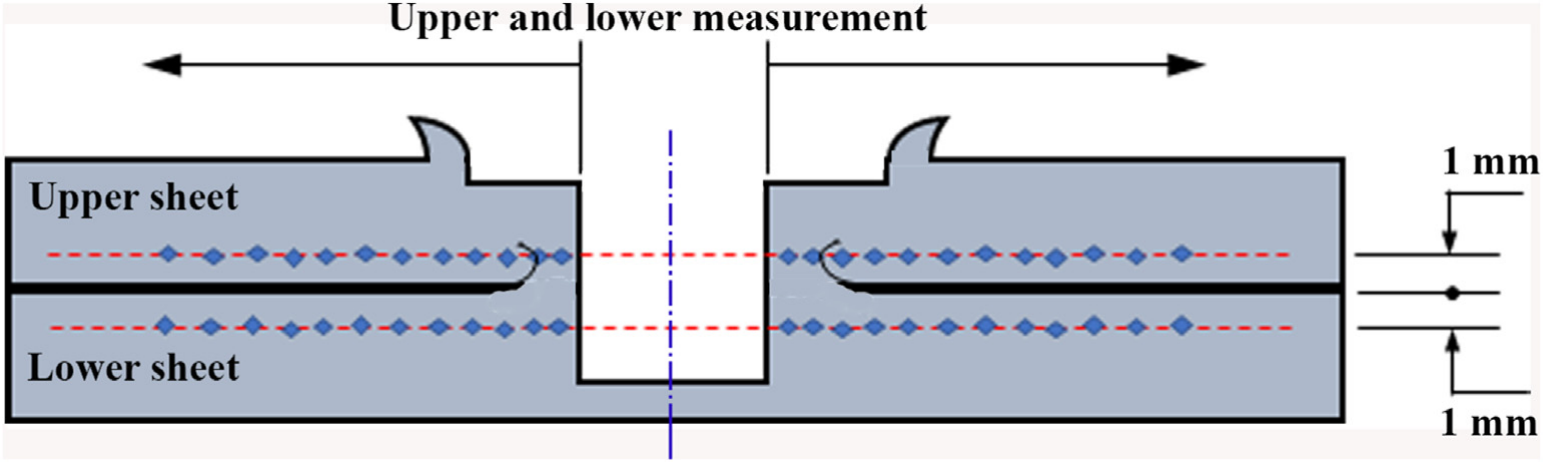
Position of hardness measurement [36].

Hardness measurement was conducted within the joint area consisting of SZ, TMAZ, HAZ, and BM as depicted in Figure 4. The Vickers microhardness was measured by using Buehler testing machines. The testing procedure referred to the ASTM standard E384 - 73 concerning the standard test method for microhardness of materials. Vickers microhardness profile was determined on both cross-sections of upper and lower sheets. The distance between the adjacent indentations was 0.5 mm, and with a load of 100 g and a dwell time of 10 s.

**Figure 5:**
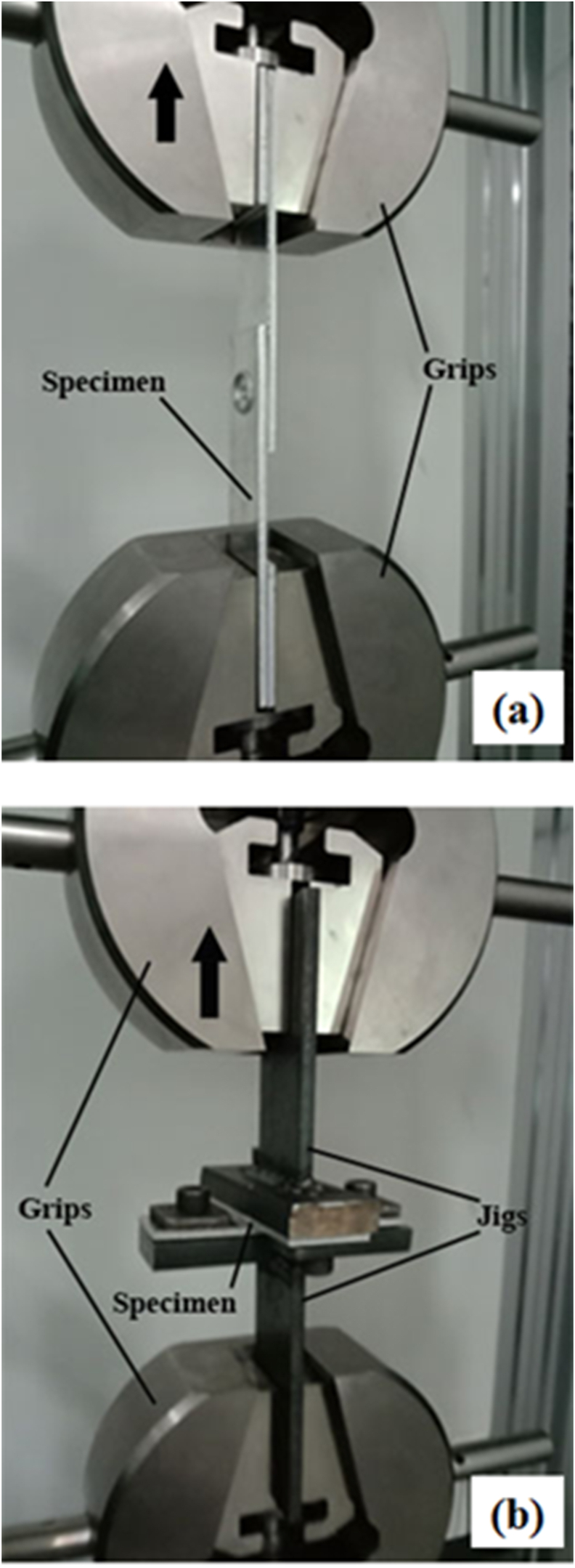
Photographs of: (a) Tensile/shear test. (b) Cross-tension specimen test.

The strength of the welded joints was determined through tensile/shear and cross-tension tests. For the tensile/shear test, the direction of loading was parallel to the interface plane. The tensile/shear and cross-tension tests were done following ISO 14273 and ISO 14272 standards, respectively. For the cross-tension test, the loading was perpendicular to the cross-section of the joint. Both tests were conducted on a universal testing machine (Shimadzu Servopulser) at a fixed cross head speed of 10 mm/min and at a load of 2000 kg. All the tensile/shear and cross-tension strengths were gained by averaging the strengths of three specimens. The schematic illustrations of both tensile/shear and cross-tension tests were presented in Figures 5a and 5b, respectively.

Both macrostructure and microstructure observations were carried out on the as welded and tensile/shear tested specimens. As welded and tested specimens were cut in the plane consisting of the rolling direction of the sheet. All specimens were prepared following standard metallographic procedures such as grinding, polishing, and etching. For macrostructure examinations, specimens were etched with the Poulton's solution (60 mL HCl, 30 mL HNO_3_, 5 mL HF, and 5 mL H_2_O). For microstructure examinations, specimens were etched by the Keller's reagent (2.5 mL HNO_3_, 1.5 mL HCl, 1.0 mL HF, and 95 mL H_2_O. Both macrostructures and microstructures were observed by using OLYMPUS optical microscopy.

The microstructure of the as-received specimen was also observed as a comparison. Weld geometry parameters such as hook height, width of FBR, PBR, and UBR were determined directly from macrostructure using the same optical microscopy. The fracture surface of the failed cross-tension specimens was observed using scanning electron microscopy (Thermoscientific type Quanta 250 SEM).

## 3 Results and discussion

### 3.1 Temperature profile

**Figure 6:**
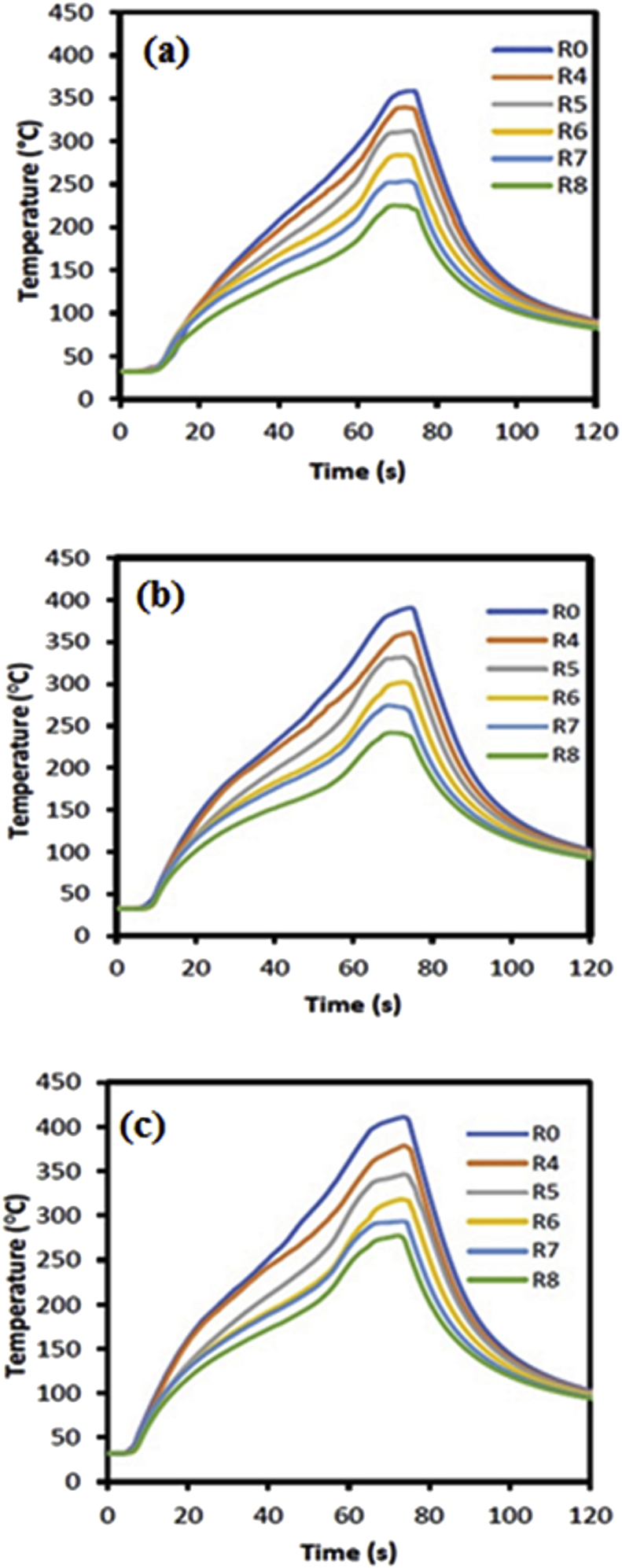
Temperature profiles of FSSW AA5052-H112 using a cylindrical pin with variations in rotational speed: (a) 900 rpm (b) 1400 rpm (c) 1800 rpm.

**Figure 7:**
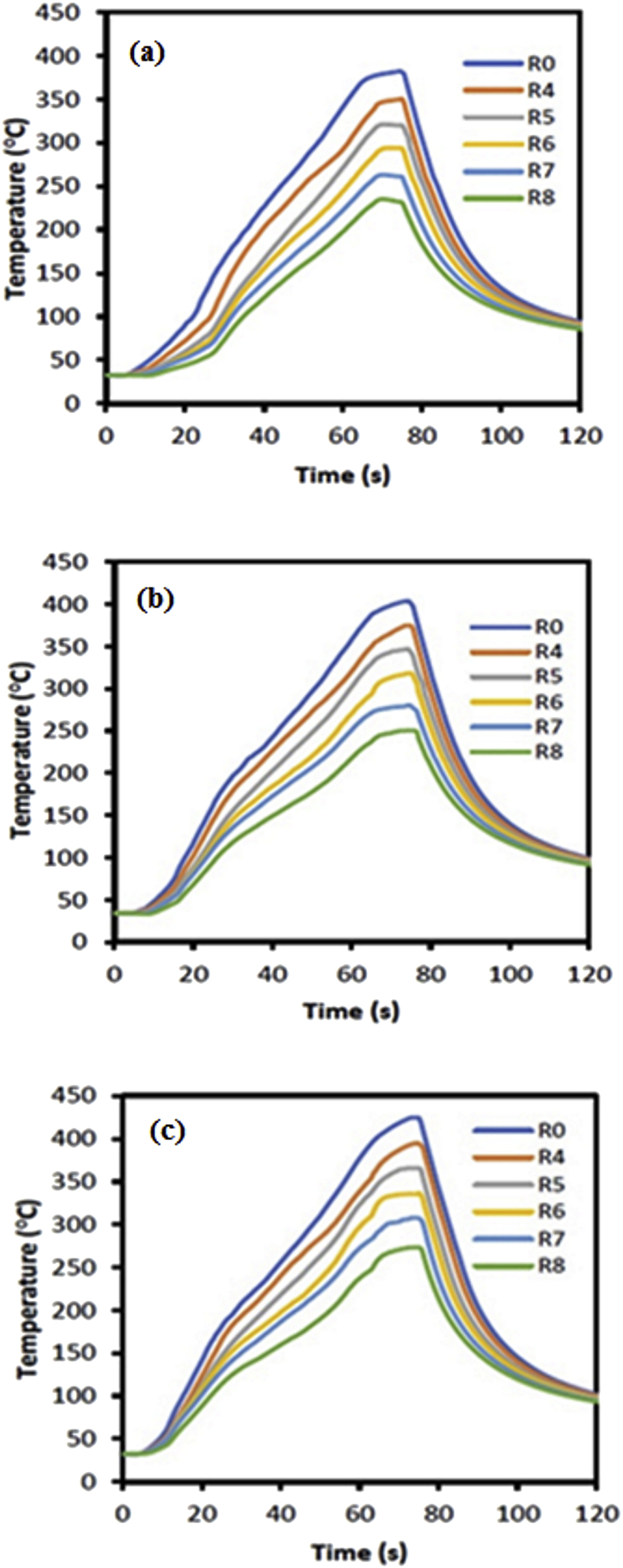
Temperature profiles of FSSW AA5052-H112 using a step pin with variations in rotational speed: (a) 900 rpm (b) 1400 rpm(c) 1800 rpm.

The temperature profiles during the FSSW AA5052-H112 process using cylindrical and step pins are shown in Figures 6 and 7, respectively. There were six observation points set, that were at the center of rotation of the tool r = 0, 4, 5, 6, 7, and 8 mm. Temperature measurement started from the tool starting to rotate, descending until it touched the workpiece surface, entering deforming material to form a welded structure, holding for a moment, until the tool was retracted from the workpiece. The temperature did not change when the tool rotated down before touching the workpiece surface. The temperature rised slightly when the tool touched the workpiece surface for some time. The temperature rised continuously and sharply until it reached a peak when the tool was in maximum depth. During dwell time, the temperature experienced a small increase and decreased when the tool was pulled out of the workpiece. The temperature continued to decrease until to room temperature.

The temperature profile described the heat history experienced by the workpiece during the FSSW process. The pin tool geometry and rotational speed affected the peak temperature reached during the FSSW process. The initial heat source was below the tool surface which was generated by the friction between the tool surface and the workpiece. The heat increased with increasing of the friction surface area and time. The heat traveled from the heat source to spread downwards and expanded in the radial direction. The highest heat was in the center of the tool and the greater distance of r the heat generated was lower.

The FSSW temperature profiles show almost the same pattern in the use of different pin geometries and rotational speeds (Figures 6 and 7). At 900 rpm rotational speed, the temperature experienced a small increase up to 60 the second, and after that, there was a sharp increase in temperature (Figures 6a and 7a). In the dwell time, the temperature experienced a small increase. The temperature started to decrease when the tool was lifted from the workpiece. At the beginning of the cooling process, the temperature decreased sharply and sloped as it approached at room temperature. There was a slight difference in the temperature profile at 1800 rpm, where was a more proportional increase from the start of the pin touching the workpiece surface until it reached the maximum depth (Figures 6c and 7c). During the dwell time and cooling, the temperature profile had the same pattern. At rotational speeds of 900, 1400, and 1800 rpm, the peak temperature reached 358, 390, and 410 °C for the cylindrical pin and 381, 403, and 424 °C for the step pin, respectively. This indicated that the peak temperature increased by increasing rotational speed for both cylindrical and step pins. This was due to the increase in rotation increased the frictional heat which in turn resulting in higher peak temperatures. Moreover, this then led to an increase in the stir zone area [15]. These results were consistent with previous results reported by Li et al. [37].

### 3.2 Macrostructure observation

**Figure 8:**
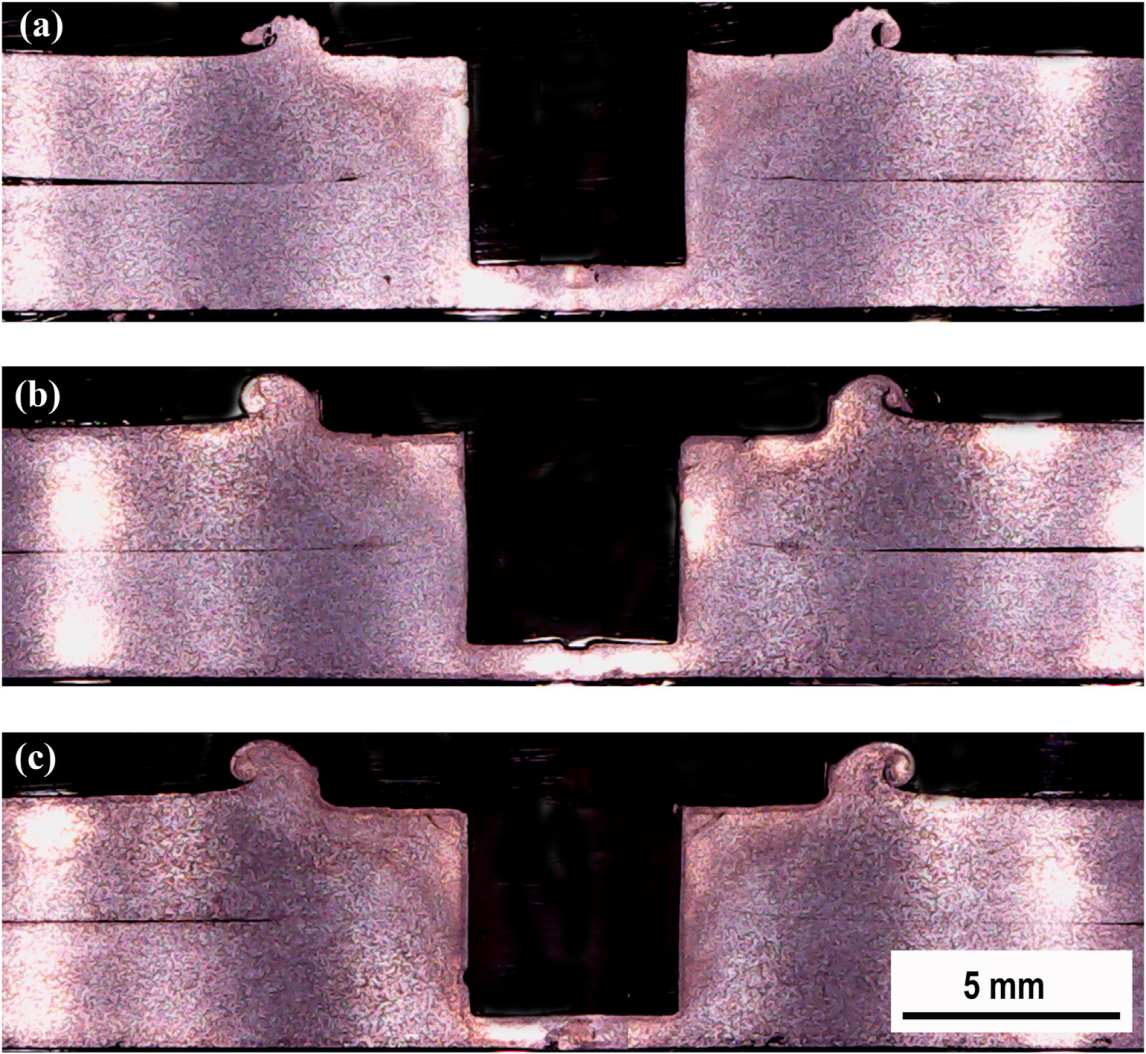
Cross-sectional macrostructure of the FSSW joint welded using cylindrical pin under different rotational speeds: (a) 900(b) 1400(c) 1800 rpm.

**Figure 9:**
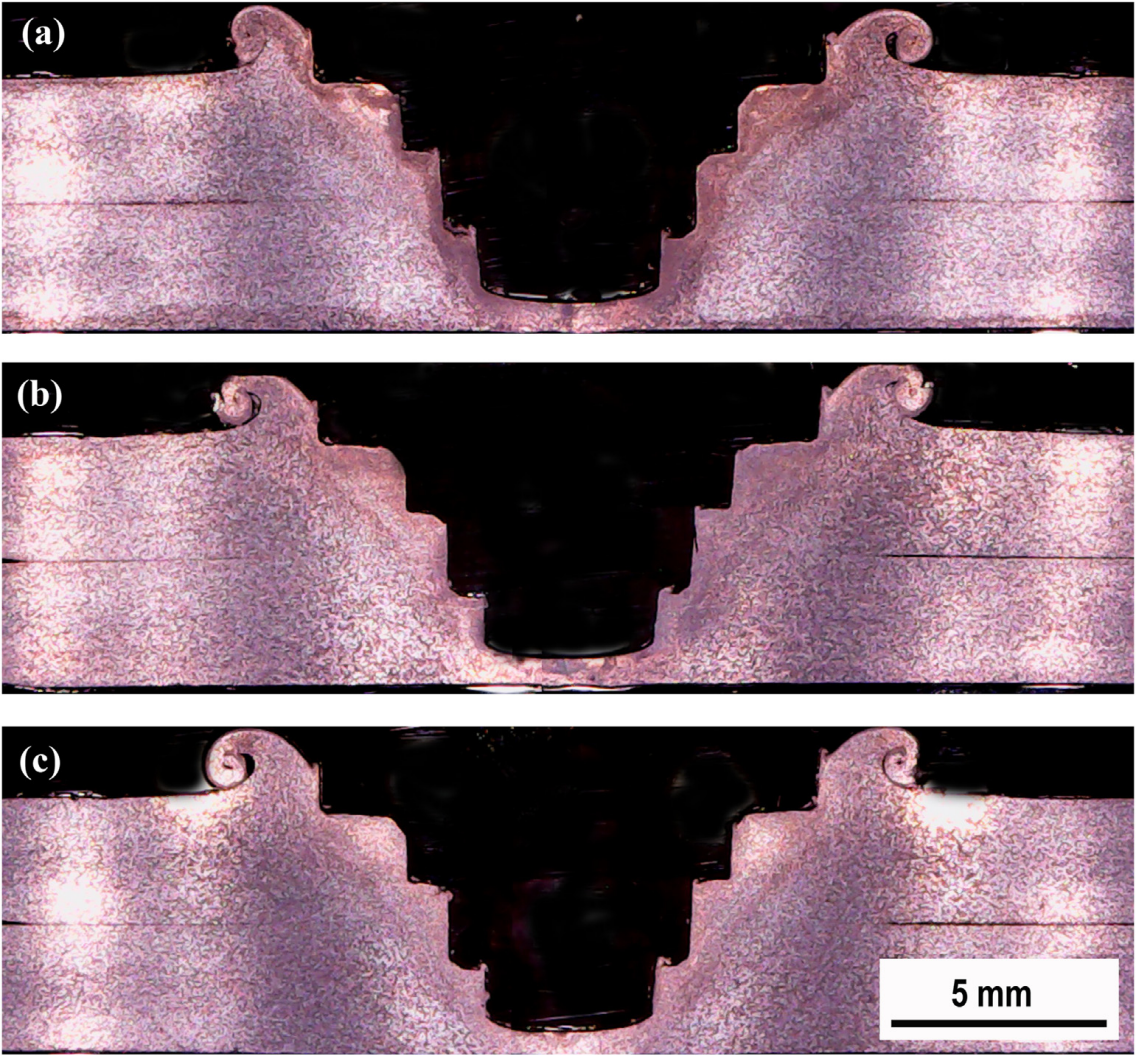
Cross-sectional macrostructure of the FSSW joint welded using step pin under different rotational speeds: (a). 900 (b). 1400 (c). 1800 rpm.

**Figure 10:**
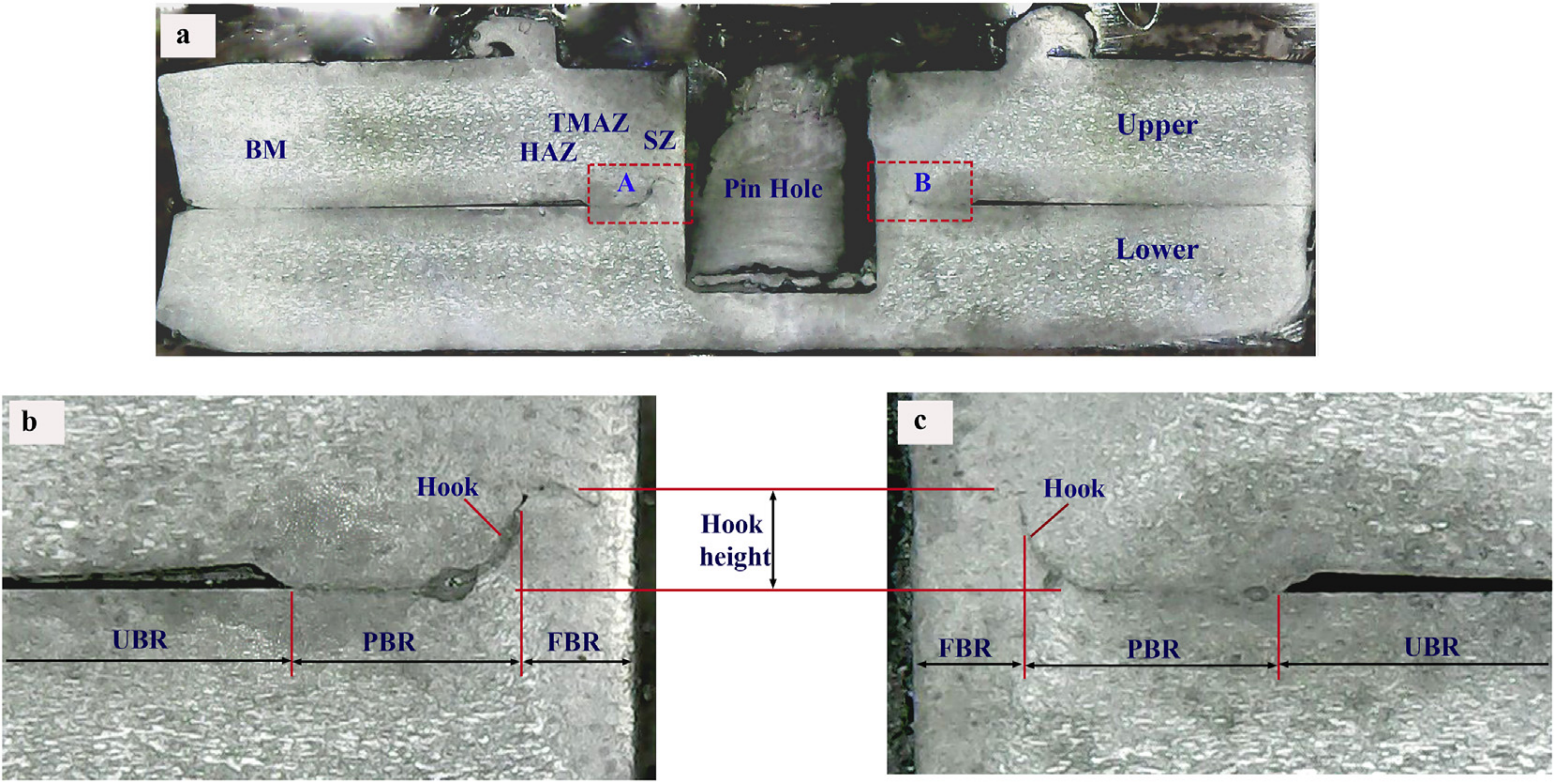
(a) Cross-sectional macrostructure of the selected FSSW joint, and (b, c) magnified views of the region A and B marked in (a).

Macrostructure observation was conducted on the cross-section of the FSSW joint. A comparison of the cross-sectional macrostructure of the FSSW joints using the cylindrical pin and the step pin at 900, 1400, and 1800 rpm are displayed in Figures 8 and 9, respectively. Furthermore, the characteristic of the cross-section of FSSW joint was classified by four zones, namely the base material (BM), heat affected zone (HAZ), thermo-mechanically affected zone (TMAZ), and stir zone (SZ) as presented in Figure 10a. The cross-section of the FSSW joint in detail is also presented in Figures 10b and 10c. Figures 10b and 10c display the magnified view of regions A and B marked in Figure 10a, respectively. The weld structure pattern was symmetrical with the tool axis which had a special characteristic that there was a hole formed by the tool pin. Bonds at the interface can be divided into three criteria, that are fully bonded region (FBR), partially bonded region (PBR), and unbonded region (UBR) [38]. FBR was characterized by the loss of top and bottom sheet interfaces, forming perfect bond due to stirring. PBR was still visible in the upper and lower sheet interface lines. The bond formed was due to the diffusion process between the top and bottom sheet surfaces. The partial bonding was indicated by the discontinued interface line. UBR was characterized by the space between the upper and lower sheets that separate the two surfaces. There was no bonding on the upper and lower sheet surfaces.

During the FSSW process, the tool rotated while pressing down, causing the material around the pin to stir and deformed simultaneously. The stirring and deformation process resulted in the movement of material flowing in a circle upward to form a spiral. The material flow was capable of breaking and cutting the interface lines around the pin to a certain distance.

At the end of the process, in this area, there was full metallurgical bond between the upper and lower sheets. The bond in this area was called the FBR. The stirring and deformation process moved the material flowing spiraling upward resulting in bending of the interface line upward and breaking off at a certain distance. After that, the interface line bent upward to form a radius, which was caused by the deformed bottom plate material pressing the top plate material. In this area the mixing has not yet occurred, there was still lines separating the upper and lower plate material. Interface lines were still clearly visible forming like a mountain and this area was called a hook. Bonding in this area was not perfect and then was called PBR. The hook height represents the spacing between the interface of two sheets and the position at which partial metallurgical bonding begins [39].

**Figure 11:**
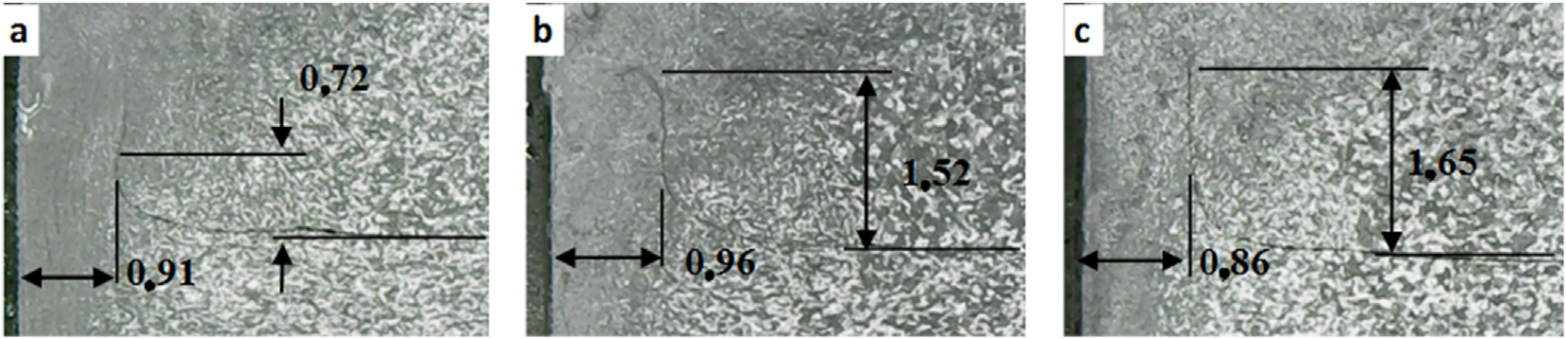
FBR width and hook height on FSSW AA5052-H112 joint welded using a cylindrical pin under different rotational speeds: (a). 900 (b). 1400 (c). 1800 rpm.

**Figure 12:**
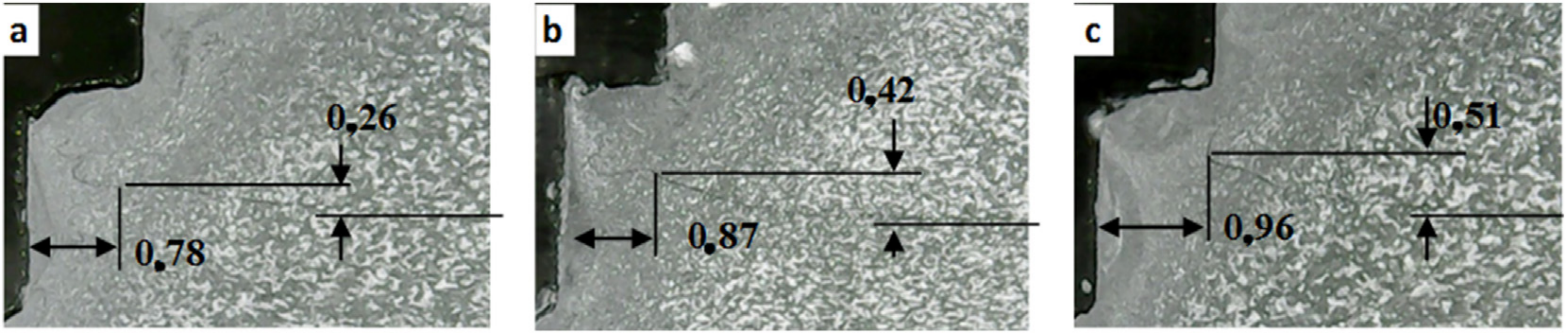
FBR width and hook height on FSSW AA5052-H112 joint welded using a step pin under different rotational speeds: (a). 900 (b). 1400 (c). 1800 rpm.

The FBR width and hook height as a function of rotational speed for the cylindrical pin is presented in Figure 11. It was found that the FBR width of the joints varies at different tool rotational speed. The width of the FBR is 0.91, 0.96, and 0.86 mm at different tool rotational speeds of 900, 1400, and 1800 rpm, respectively. This indicated that the width of FBR enlarged with increasing the rotational speed up to 1400 and then decreased at 1800 rpm. The maximum in the width of the FBR was achieved at 1400 rpm. Similar findings were also reported by Mahmoud and Khalifa [34] where with increasing the rotational speed up to 800 rpm increased the FBR width and further increase reduced the FBR width of the FSSW AA 5754 aluminum alloy. From Figure 11, it was also found that the hook height was 0.72, 1.52, and 1.65 mm for the rotational speeds of 900, 1400, and 1800 rpm, respectively. This suggested the hook height increased as the rotational speed increased. In other words, the maximum value of the hook height was obtained at the highest rotational speed. Furthermore, the effect of the rotational speed on the FBR width and hook height of the joints for the step pin is demonstrated in Figure 12. It was found that the FBR width was 0.78, 0.87, and 0.96 at the rotational speeds of 900, 1400, and 1800 rpm, respectively. This indicated that the FBR width increased as the rotational speed increased. Moreover, the hook height obtained at 900, 1400, and 1800 rpm was 0.26, 0.42, and 0.51 mm, respectively. This indicated the hook height increased with increasing the rotational speed. This trend was similar to the FBR width. Thus, for the step pin, the FBR width and hook height increased with increasing rotational speed. This was probably related to the increased frictional heat generated and the expansion of the stirred interface zone [34]. As shown in Figures 11 and 12, the FBR width for the cylindrical pin at 900 and 1400 rpm was higher than that of the step pin. However, the FBR width of the step pin at 1800 rpm was higher than that of the cylindrical pin. Besides that, the cylindrical pin exhibited a higher hook height at all rotational speeds compared to the step pin. The variations of both FBR width and hook height obtained under different types of pin geometry and rotational speed will influence the strength of FSSW joints and will be discussed later.

### 3.3 Microstructure characterization

**Figure 13:**
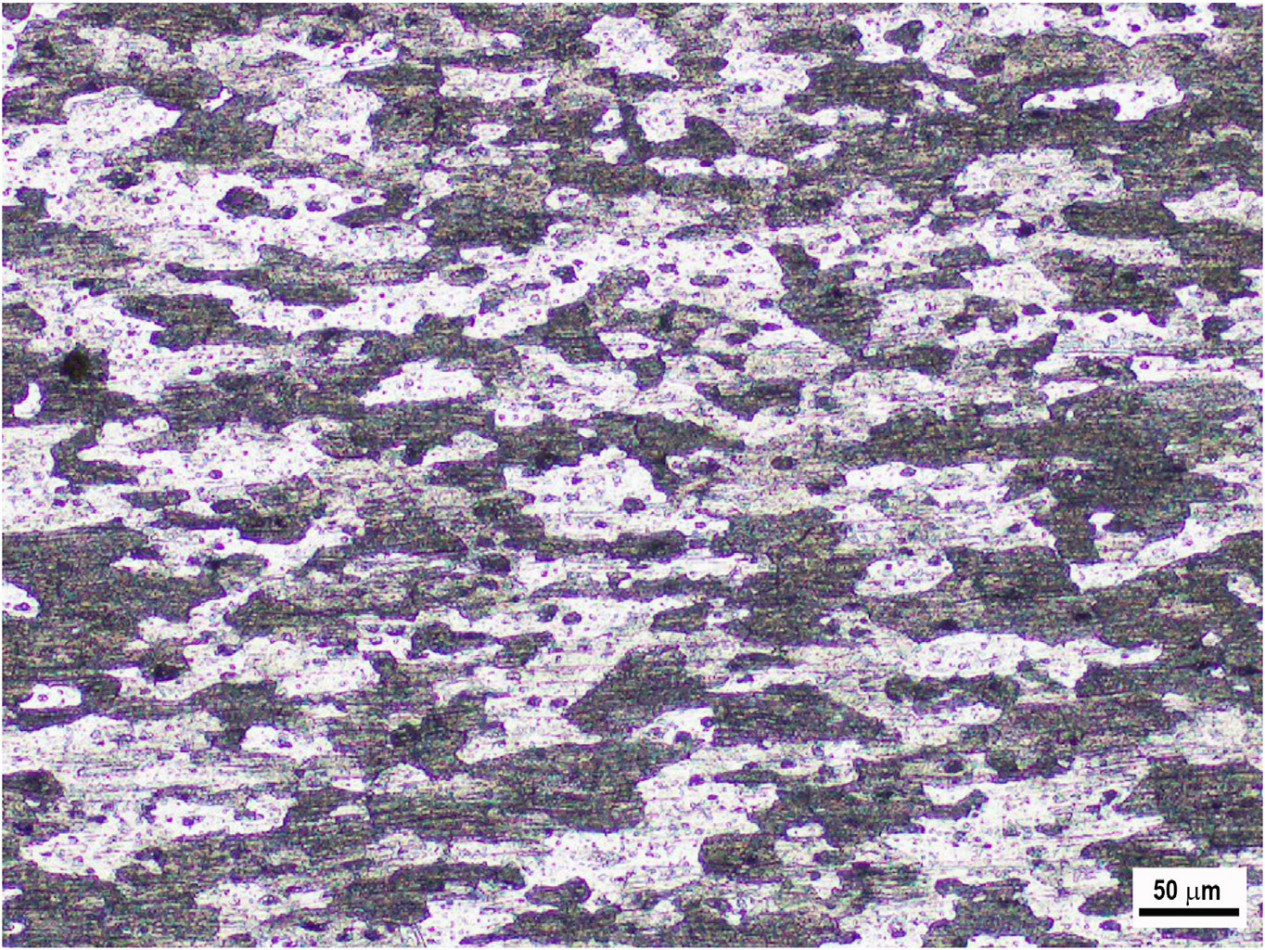
Cross-sectional microstructure of base metal of AA 5052-H112 aluminum alloy.

**Figure 14:**
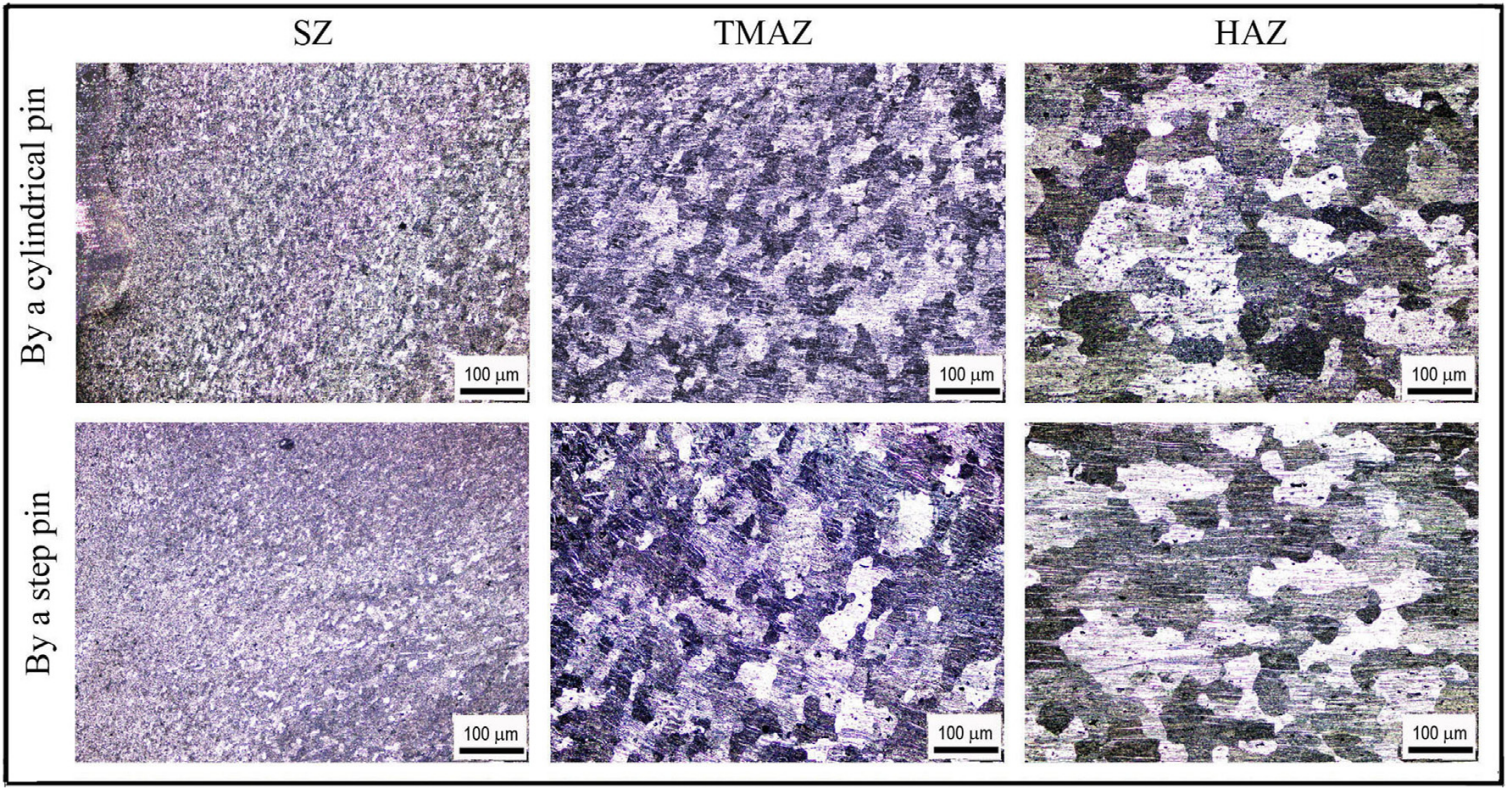
Cross-sectional microstructures of welds made using both cylindrical and step pins at 1400 rpm.

Figure 13 displays the microstructure of the base material (BM). The elongated grains and second-particle phases parallel to the rolling direction was observed. Figure 14 depicts the cross-sectional microstructure of the SZ, TMAZ, and HAZ prepared at 1400 rpm using both cylindrical and step pins. The more refined and equiaxed grains were exhibited in the SZ compared to that of TMAZ and HAZ for both the cylindrical and step pins. Both the SZ and TMAZ exhibited smaller grains than HAZ. The refined grains in both the SZ and TMAZ might be associated with more plastic deformation and higher temperatures during pin stirring and a friction thermal cycle that caused the microstructure to undergo dynamic recrystallization, resulting in finer and homogeneous grains [14]. On the other hand, the coarser grains were shown in the HAZ microstructure. This was due to the HAZ only experienced friction heating without plastic deformation [14]. From Figure 14, there were similar microstructures of SZ, TMAZ, and HZ by using both cylindrical and step pins. It can be concluded that the pin geometry did not significantly affect the microstructure of FSSW welds.

### 3.4 Hardness profiles

**Figure 15:**
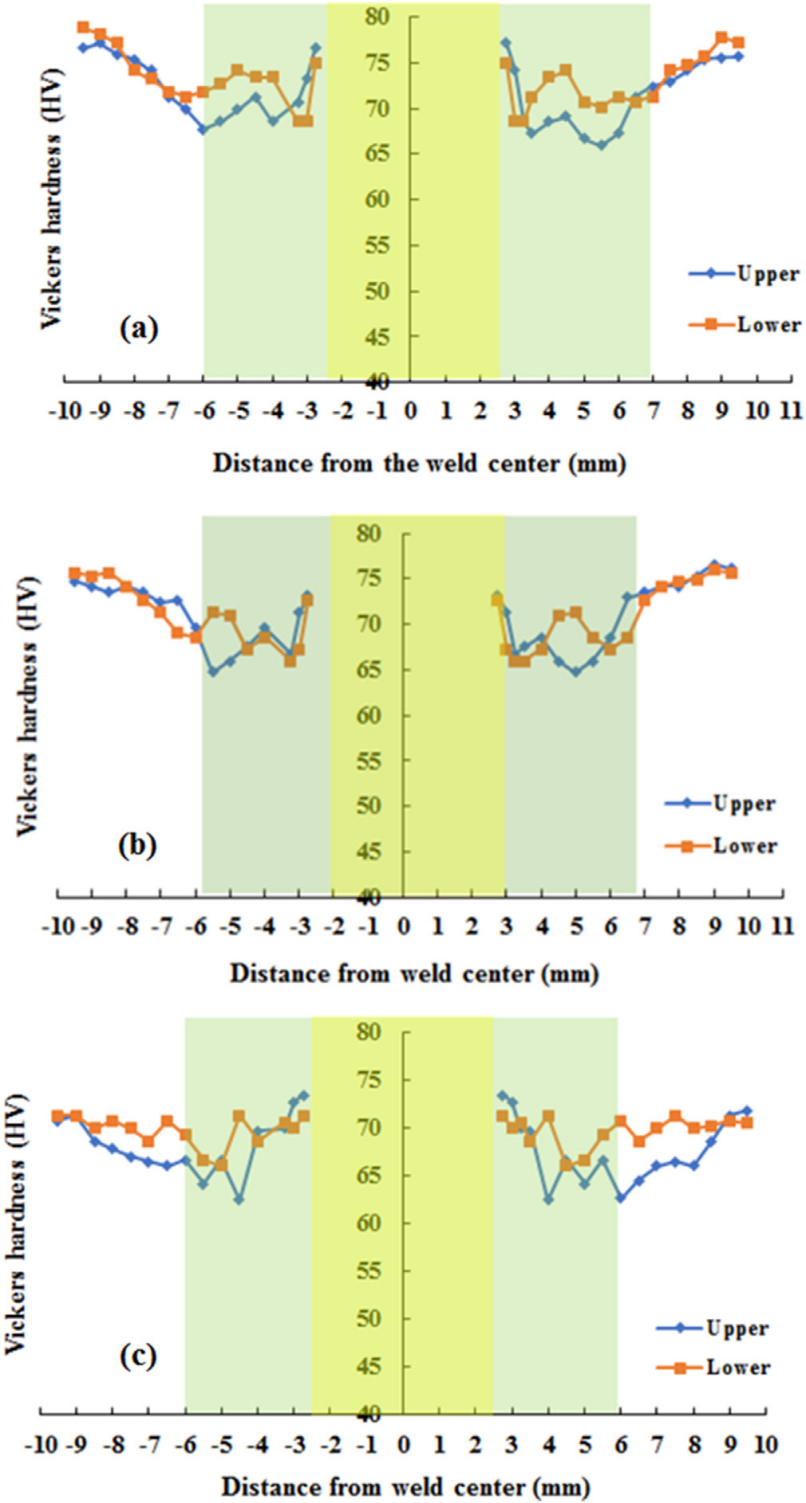
Hardness distributions of the welds made with cylindrical pin at various rotational speeds: (a). 900 (b). 1400 (c). 1800 rpm.

**Figure 16:**
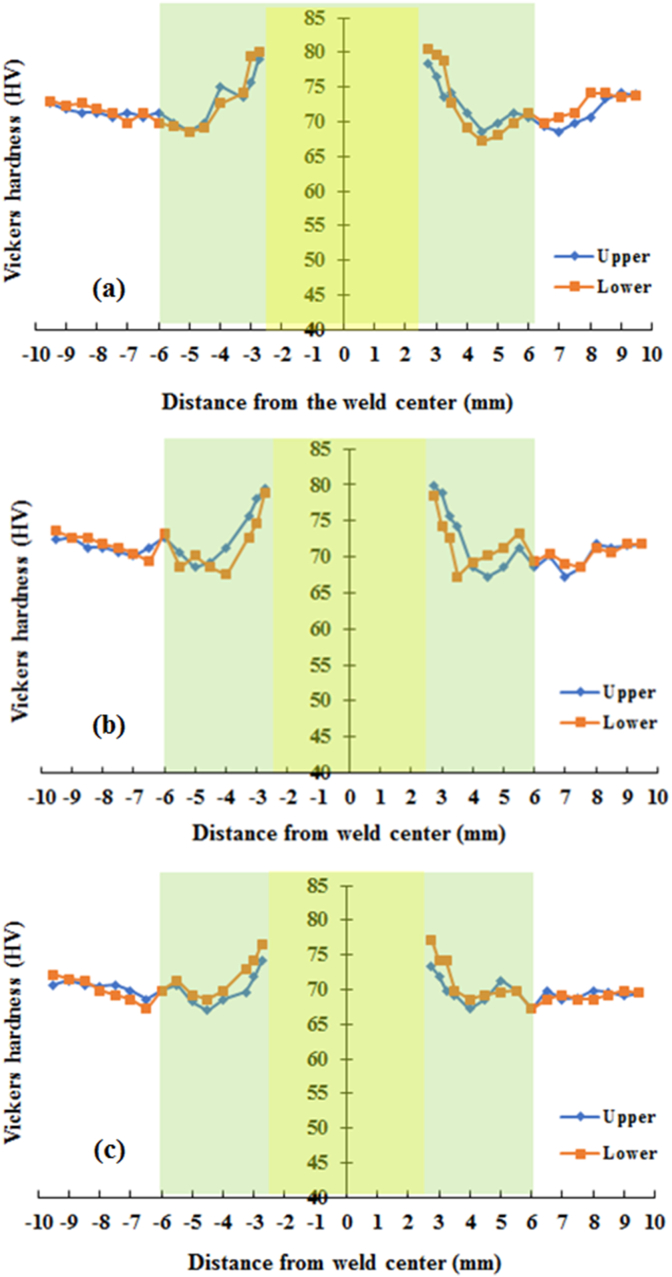
Hardness distributions of the welds made with a step pin at various rotational speeds: (a). 900 (b). 1400 (c). 1800 rpm.

The Vickers hardness measurement was carried out in the middle of the thickness of the upper and lower sheets. The measurements were made in the horizontal direction which was 1 mm away from the interface that started from the side of the keyhole. The Vickers microhardness profiles on the cross-section of the joints welded using both cylindrical and step pins at various rotational speeds are presented in Figures 15 and 16, respectively. The Vickers hardness distribution for the cylinder and step pins was obtained to be symmetrical with respect to the center of the welding keyhole at all rotational speeds, showing the W-shaped view. In general, both upper and lower sheets had almost the same Vickers hardness on the welds using both cylindrical and step pins. From Figure 15, it can be also seen that the welds made with the cylindrical pin exhibited a slightly lower hardness in the stir zone than in the base metal. The higher hardness was shown in the stir zone than in both TMAZ and HZ. This was ascribed from a finer grain in the stir zone as presented in Figure 14. From Figures 15 and 16, for both cylindrical and step pins, the hardness of the weld reduced slightly with an increase of the rotational speed. This might be associated with coarsening size in the stir zone [34, 40, 41]. A slight decrease in the hardness of the welds suggested that the rotational speed had no obvious effect on the hardness of the welds. These were in consistent with the results reported by previous researchers [14, 34]. Furthermore, at 1400 rpm, the stir zone close to the keyhole welded using the cylindrical pin exhibited the hardness of 74 HV whereas one welded using step pin had the hardness of 80 HV. This indicated that only slight differences in the hardness were shown by both types of pins. In other words, the pin geometry did not affect the hardness of the welds. Figure 16 exhibits the profiles of the hardness of the welds made with the step pin. At all rotational speeds, it was found that a higher hardness was exhibited in the SZ than in the base metal. A similar finding was demonstrated by Bozzi et al. [16] where the hardness of AA5182 FSSW welds was higher than that of the BM. The higher hardness in the SZ than in the base metal was again related to a finer grain in the SZ as displayed in Figure 14. According to Hall-Petch equation [40, 41], the metal materials with smaller grain sizes have greater yield strength and hardness. The hardness decreased in both HAZ and TMAZ regions and subsequently increased in the SZ toward the direction of the keyhole. The reduction in hardness of both HAZ and TMAZ was probably associated with the coarsening in grain sizes during the weld thermal cycle as shown in Figure 14 [27]. Furthermore, a higher hardness in the SZ compared to that of TMAZ and HAZ was again be attributed to finer grain structure as depicted in Figure 14 based on the Hall-Petch equation [40, 41]. It was concluded that the hardness of the welds was slightly influenced by both rotational speed and pin.

### 3.5 Tensile/shear and cross-tension loads

**Figure 17:**
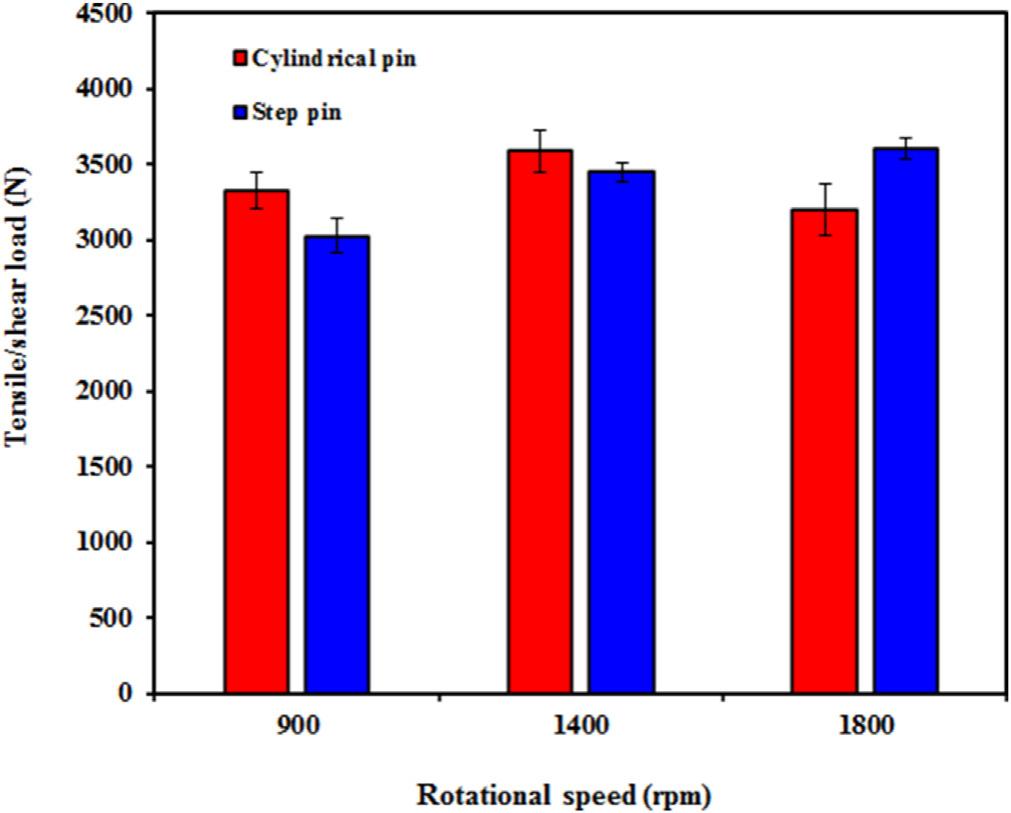
Tensile/shear load of FSSW AA5052-H112 welds as a function of both geometry pin and rotational speed.

The tensile/shear and cross-tension loads of the FSSW AA5052-H112 joints were determined through the tensile/shear (lap shear) and cross-tension tests, respectively. The effects of both pin geometry and rotational speed on the tensile/shear load of the welds are presented in Figure 17. At the rotational speeds of both 900 and 1400 rpm, the joints welded using the cylindrical pin exhibited a higher tensile/shear load than the step pin. This was attributed to higher FBR width in the joint using a cylindrical pin compared to step pin at speeds of 900 and 1400 rpm as presented in Figure 11. On the other hand, at 1800 rpm, the joint welded using the step pin showed a higher tensile/shear load compared to the cylindrical pin. This was associated with the higher FBR width for the step pin (0.96 mm) than the cylindrical pin (0.86 mm) as demonstrated in Figure 12. Furthermore, for the cylindrical pin, the tensile/shear load increased slightly with increasing rotational speed from 900 to 1400 rpm but then decreased at 1800 rpm. The tensile/shear load achieved a maximum value of 3589 N at 1400 rpm. The increase in rotational speed from 900 to 1400 rpm caused higher frictional heat and more intensive flow of plastic material which further resulted in an increase in the width of the FBR [34, 42]. The increase in the width of the FBR increased the ability of the joint to withstand the load, resulting in a higher tensile/shear load. As shown in Figure 11, the FBR width reached a maximum value at 1400 rpm for the cylindrical pin. The maximum value of the FBR width corresponds to the tensile/shear and cross-tension load. Furthermore, for the cylindrical pin, the tensile/shear load decreased at 1800 rpm. This was associated with the excessive thinning of the upper sheet [42, 43]. Reduction in stir zone size at higher rotational speed might be also believed to be responsible for the reduced cross-tension load [44]. Moreover, the reduced tensile/shear load at 1800 rpm was also attributed to the smallest dimension of the FBR among others as presented in Figure 11. Higher rotational speed (1800 rpm) produced higher heat leading to easier movement of the material and a wider range. At high temperatures, the solid material experienced a decrease in the pressing strength gradient. Thus, the ability to compress solid materials to deform and move the material decreases resulting in a smaller FBR dimension at 1800 rpm and thus dropped tensile/shear loads. Similar findings were also demonstrated by Li et al. b where the tensile/shear strength of FSSW 2A12-T4 aluminum alloy increased with an increase of speed up to 1300 rpm owing to higher heat and longer diffusion time resulting in the improved tensile/shear load. However, tensile/shear loads decreased at speed over 1300 rpm caused by excessive upward bending of the hook and coarse grain along the SZ. Similar findings were also demonstrated by Bozzi et al. [16] and Tozaki et al. [45] and on the FSSW aluminum alloy. They reported that the tensile/shear failure load increased by increasing speed up to 1300 rpm and then decreased at above 1300 rpm. Increasing the rotational speed up to 1300 rpm produced more extensive stirring and higher heat that enlarged the SZ size and bonded area, and finally increased the strength of the welds. The reduced tensile/shear failure load at higher speeds was probably attributed to both the smaller stir zone and the increased tensile residual stress around the SZ [16]. On the other hand, for the step pin, the tensile/shear load increased with the increase in rotational speed. The highest tensile/shear load (3606 N) was achieved at 1800 rpm. This indicated that the rotational speed had a significant effect on the tensile/shear load of FSSW joints made with the step pin. This was probably ascribed from the increased FBR width where the FBR width increased with the increase in rotational speed as demonstrated in Figure 12. It was concluded that the tensile/shear load was remarkably influenced by both pin geometry and the rotational speed.

**Figure 18:**
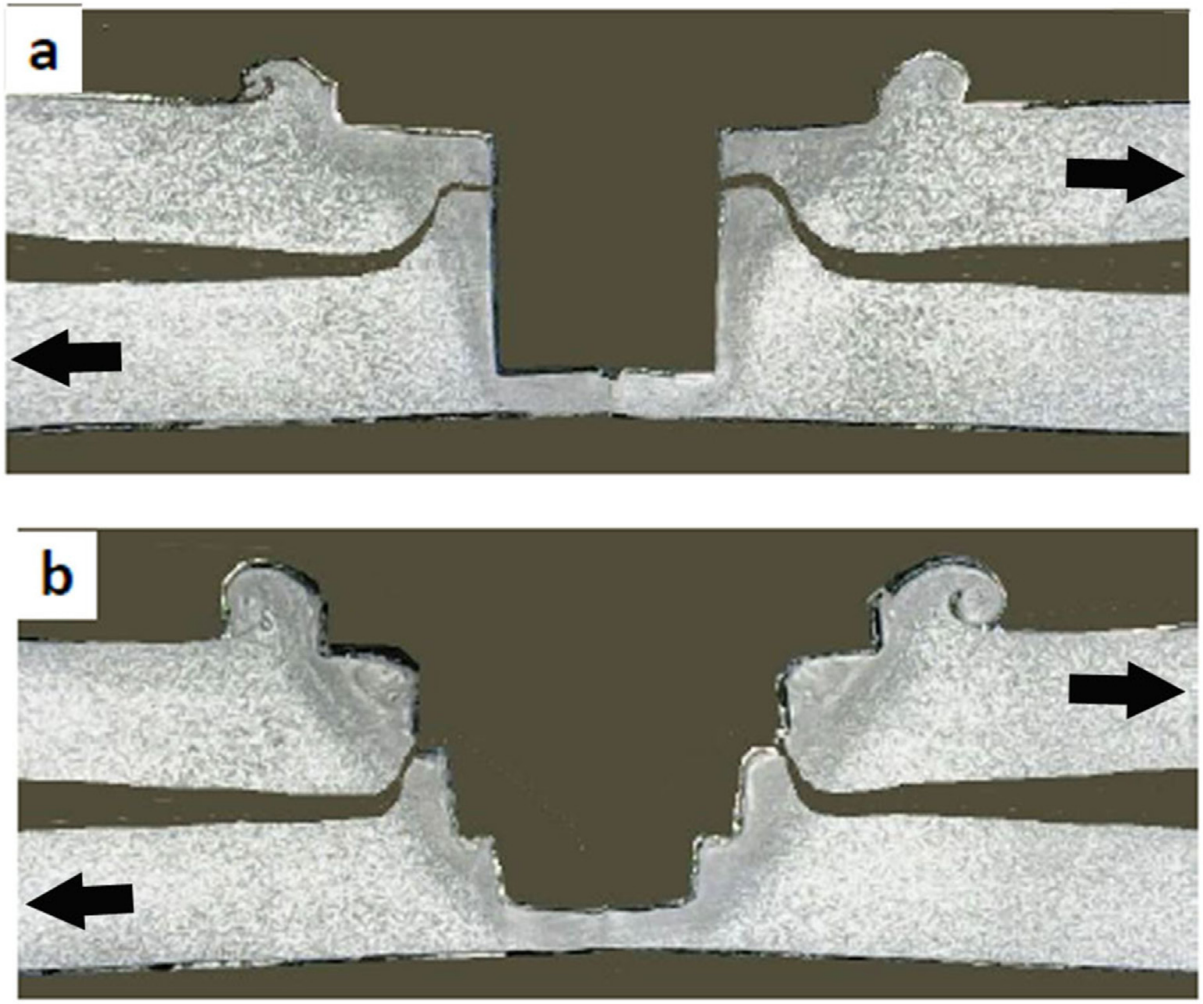
Cross-sectional macrographs of tensile/shear fractured joints using: (a) Cylindrical pin, (b) step pin.

Figure 18 displays the cross-sectional macrographs of failed joints under the tensile/shear loading using both cylindrical and step pins at 1400 rpm. The arrow indicated the loading direction of the tensile/shear test. For a cylindrical pin, the crack was started from the interface of the lower and upper sheets especially started from un-bonded region and then crack propagated through the hook's path in the lower sheet at the stir zone/TMAZ interface. The final fracture occurred in the keyhole, resulting in the shear fracture as shown in Figure 18a. On other hand, for a step pin, the crack initiated from un-bonded region, propagated along partial bonded region, full bonded region, and then propagated upwards. The final fracture appeared in the upper sheet, producing the tensile/shear fracture as shown in Figure 18b. From Figure 18, it can be observed that the crack length of weld joint using a cylindrical pin was longer than that of a step pin. This confirmed the higher tensile/shear load of weld joints using a cylindrical pin was higher than that using a step pin as presented in Figure 17.

**Figure 19:**
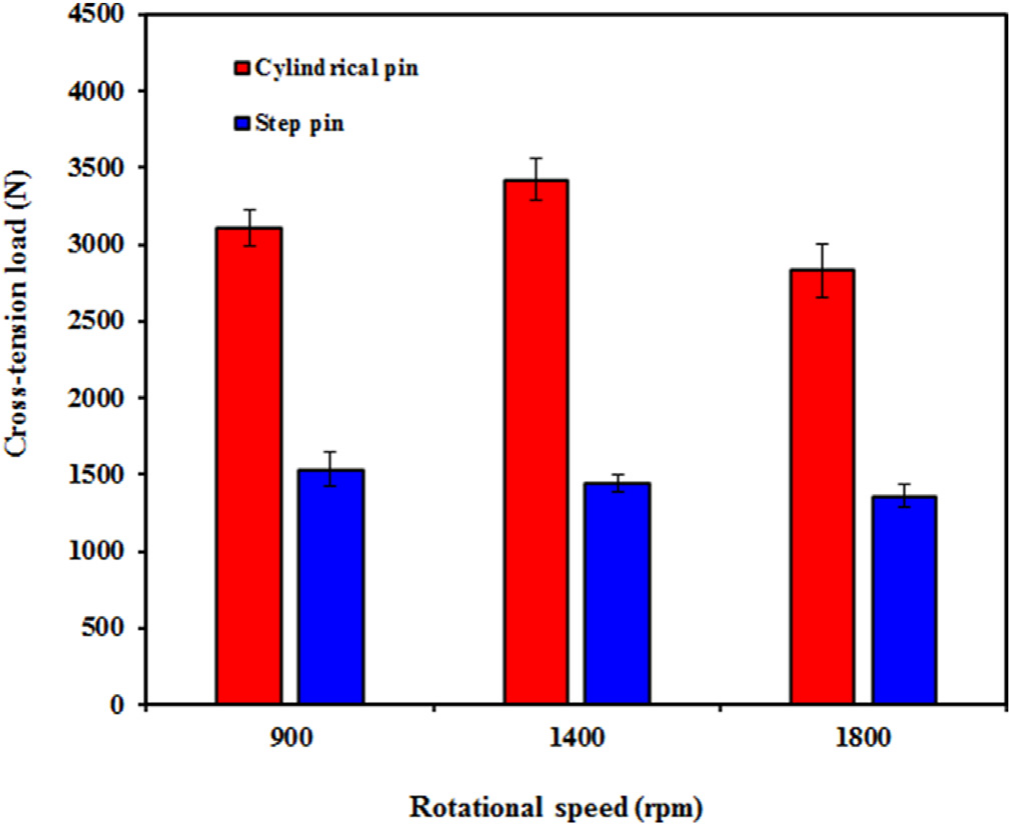
Cross-tension load of FSSW AA5052-H112 as a function of both geometry pin and rotational speed.

The cross-tension load of the welds as a function of both geometry pin and rotational speed is depicted in Figure 19. At all rotational speeds, it was found that the cross-tension load of the joints welded using the cylindrical pin was much higher than that of the step pin. This was probably attributed to both higher values in hook height and FBR width for the cylindrical pin compared to the step pin as shown in Figures 12 and 13. This also suggested that the cross-tension strength was remarkably affected by pin geometry [27, 46]. It was also reported in the literatures that the static strength of the FSSW joints was remarkably influenced by the hook geometry [27] and stir zone area [45]. In addition, Shen et al. [39] also reported that a larger effective weld width produced stronger welds. From Figure 17, it can be also seen that for the cylindrical pin, the cross-tension load firstly increased as the rotational speed increased from 900 to 1400 rpm, and then decreased at 1800 rpm. Thus, the cross-tension load reached a maximum value of 3419 N at 1400 rpm with an increase of 10%. The highest cross-tension load obtained at 1400 rpm for the cylindrical pin might be related to the largest FBR width as shown in Figure 11 among all rotational speeds. The lowest FBR width at 1800 rpm was probably believed to be responsible for the reduced cross-tension load. From Figure 19, for the step pin, the cross-tension load decreased slightly with increasing rotational speed. As presented in Figure 12, the hook height and FBR width increased with increasing rotational speeds for the joints made with the step pin. However, for the step pin, the increased both hook height and FBR width unchanged remarkable the cross-tension load. This suggested that the cross-tension load of the joints welded with the step pin was not significantly affected by the hook geometry and rotational speed. Similar findings were also reported by Shen et al. [46] where there was no relationship between the hook geometry and the cross-tension loads of the FSSW AA 6061-T4 aluminum alloy. The opposite findings were demonstrated by Badarinarayan et al. [27] where the cross-tension strength was obviously affected by the hook geometry. From Figures 17 and 18, it could be concluded that the strength of the welds was influenced by both pin geometry and rotational speed.

### 3.6 Fracture modes

**Figure 20:**
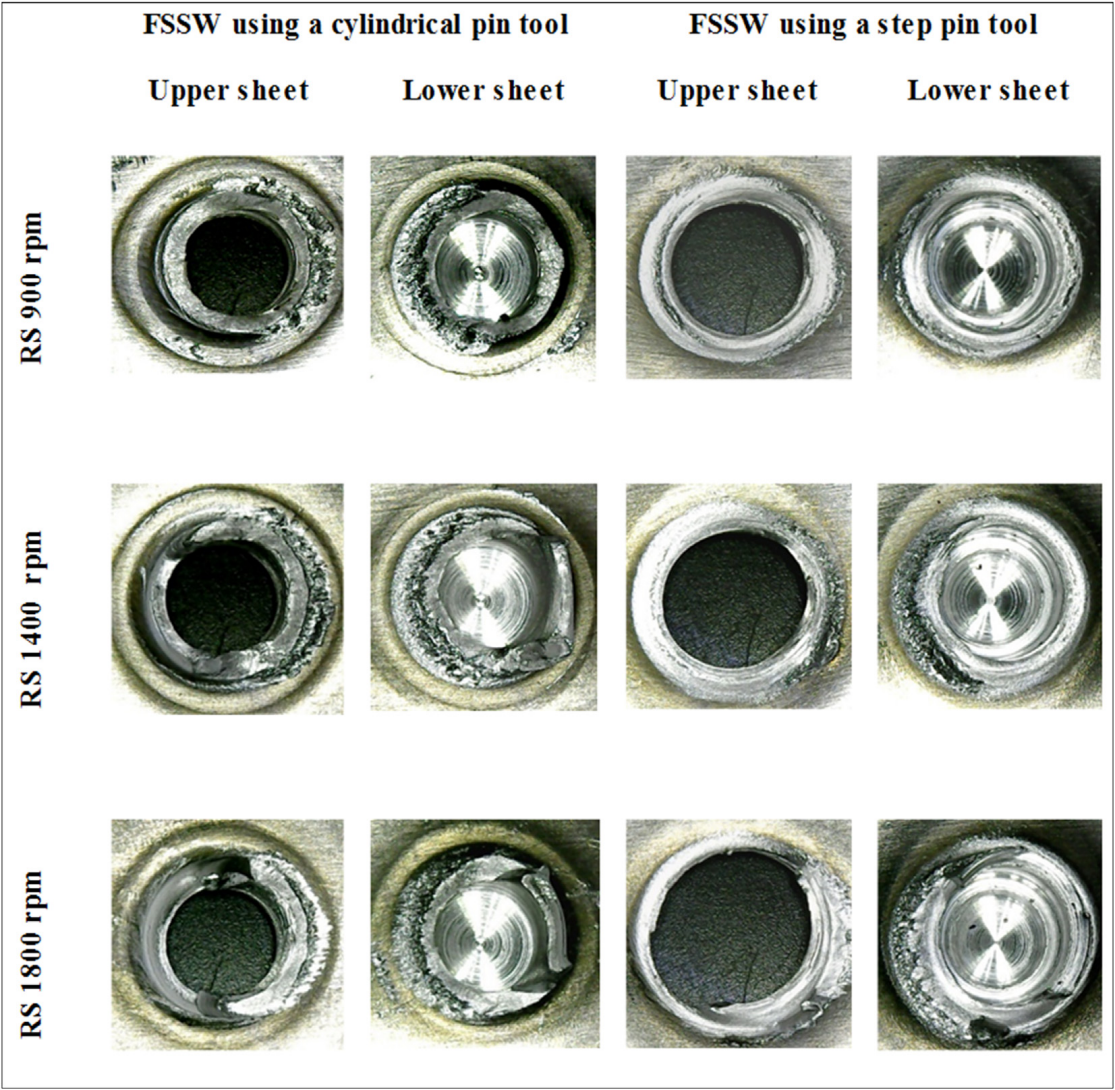
Macrographs of the tensile/shear fractured joints using both cylindrical and step pins at different rotational speeds.

Figure 20 displays the macrographs of the tensile/shear fractured joints for both cylindrical and step pins at different rotational speeds. For each geometry pin, the left figures exhibit the upper sheets observed from the bottom whereas the right figures exhibit the lower sheets observed from the top. For welds made using cylindrical pins, all joints failed in shear fractures as indicated by the fractures that occurred between the upper sheet and the loading side of the lower sheet. It was also found that increasing rotational speed did not affect the fracture mode for a cylindrical pin. The shear fracture was found in the welds made with the cylindrical pin at all rotational speeds. On the other hand, for the step pin, mixed tensile/shear fracture was observed at all rotational speeds. The tensile fracture first took place at the position with the thinner thickness of the upper sheet, then propagated along the circumferential direction and the shear fracture finally occurred. Furthermore, the diameter of the button left in the upper sheet became larger by increasing rotational speed. Similar failure modes were also demonstrated by Fujimoto et al. [47], Tozaki et al. [48], and Yoon et al. [49]. From Figure 20, it was concluded that the fracture mode under tensile/shear loading was influenced by the pin geometry but not the rotational speed.

**Figure 21:**
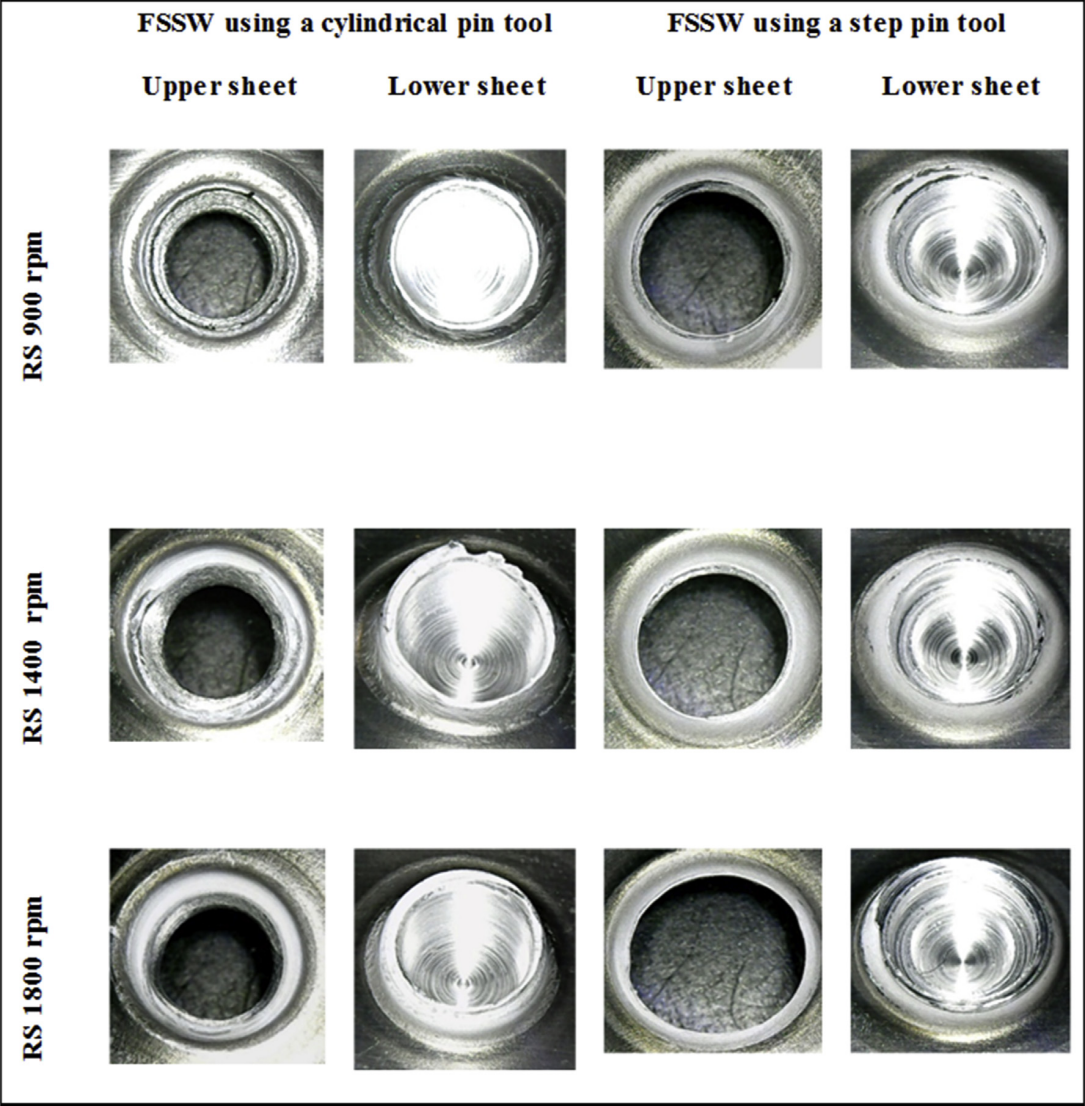
Macrographs of the cross-tension fractured joints using both cylindrical and step pins at different rotational speeds.

Figure 21 displays the macrographs of the cross-tension fractured joints using both cylindrical and step pins at different rotational speeds. For a cylindrical pin at all rotational speeds, the failure mode of nugget debonding under cross-tension load was observed as shown by the faying surface between the upper and lower tearing sheets. On the other hand, the nugget pull-out fracture was exhibited by all joints made using a step pin at all rotational speeds under cross-tension loading. Nugget pull-out occurred along the circumference of the thinner thickness position of the upper sheet. From Figure 21, it could be concluded that under cross-tension loading, the two typical failure modes were examined, namely nugget debonding fracture found in joints using a cylindrical pin and nugget pull-out using a step pin. The fracture mode was not influenced by increasing rotational speed but was affected by the pin geometry. Two typical failure modes were also reported by Tozaki et al. [45] and Zhang et al. [14]. However, there are differences in the failure mode between these literatures and the present study. Tozaki et al. [45] reported that the fracture of nugget debonding occurred at the lower rotational speed while the fracture of nugget pull-out at the higher rotational speed. In contrast, Zhang et al. [14] observed that the nugget debonding occurred at higher rotational speed, whereas the nugget pull-out at lower rotational speed. In the present study, under cross-tension load, the type of failure modes was influenced by the pin geometry but not by increasing rotational speed. This is consistent with the tensile/shear load.

**Figure 22:**
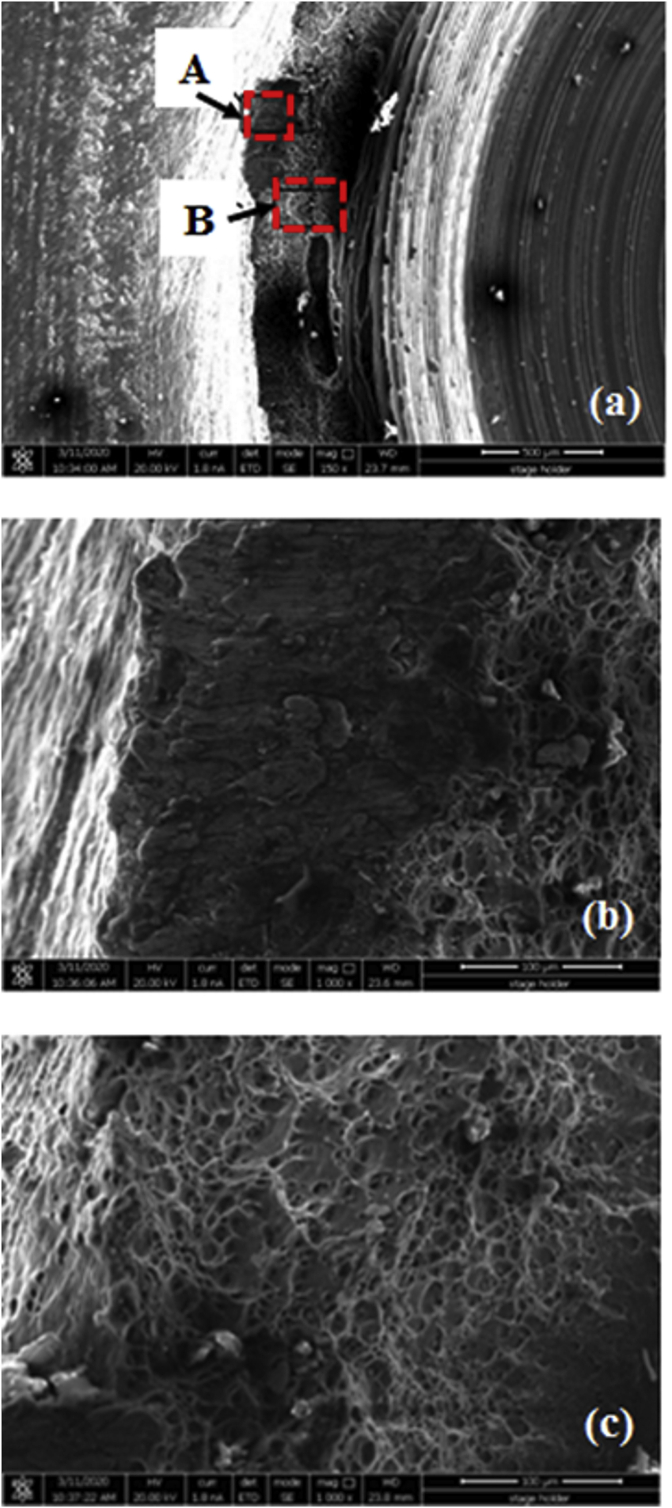
SEM images of (a) a fracture surface on the lower sheet using a cylindrical pin at 900 rpm, and (b, c) magnified views of the region A and B marked in (a).

Figure 22a depicts SEM images of the fracture surface of the failed cross-tension specimen of the lower sheet using a cylindrical pin at 900 rpm Figure 22b also displays SEM image of the fracture surface of location in the fracture surface (marked as A in Figure 22a). From Figure 22b, the rough surface and no dimple in the region A was present showing the occurrence of quasi-cleavage fracture. The region A in the outer circumference of the weld region was the final fracture. Figure 22c demonstrates SEM image of the fracture surface in the region B. There are many equiaxed dimples in area B which indicate a stronger metallurgical bond formed there. Moreover, a large of equiaxed dimples also confirmed a higher cross-tension load of the weld joints using a cylindrical pin at 900 rpm as presented in Figure 19. The crack was initiated in the hook tip and it propagated along the hook toward the surface of the upper sheet. Finally, the upper and lower sheets were separated by stir zone left in the lower sheet.

**Figure 23:**
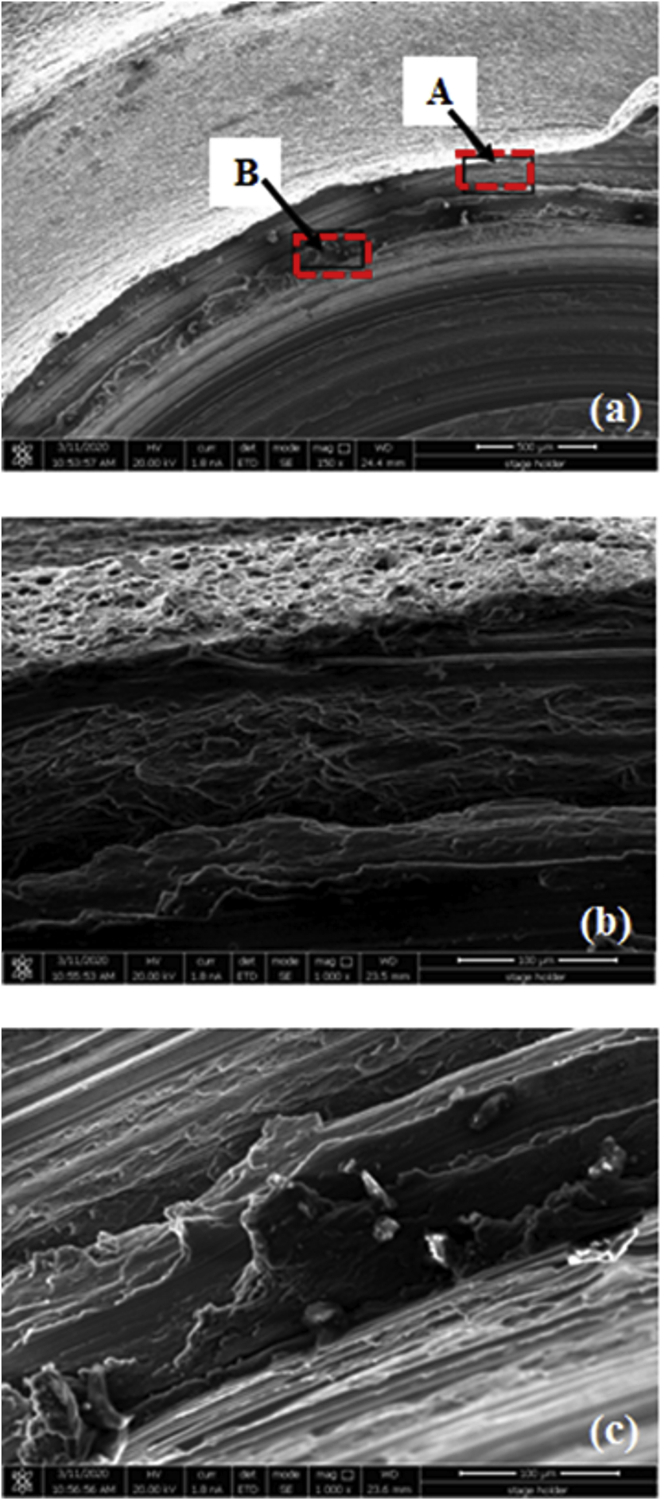
SEM images of (a) a fracture surface on the lower sheet using a step pin at 1800 rpm, and (b, c) magnified views of the region A and B marked in (a).

Figure 23a presents SEM micrographs of the overall fracture surface on the lower sheet using a step pin at 1800 rpm Figure 23b demonstrates the magnified view of region A marked in Figure 23a. The surface of region A was relatively rough, where small-elongated dimples were occurred indicating the poor-quality bonding there. In addition, small-elongated dimples were also exhibited in the region of B as shown in Figure 23c revealing poor metallurgical bonding between the upper and lower sheets. Moreover, this confirmed a lower cross-tension load of the weld joints using a step pin at 1800 rpm as shown in Figure 19 before.

## 4 Conclusions

Based on the results discussed, the conclusions can be made as follows:

• Both pin geometry and the tool rotational speed had a significant effect on the strength of the welds. At 900 and 1400 rpm, the strength of the welds made using a cylindrical was higher than that of a step pin.
• For a cylindrical pin, the tensile/shear and cross-tension loads of the welds increased with increasing rotation speed from 900 rpm to 1400 rpm and then decreased at 1800 rpm. At a rotational speed of 1400 rpm, the maximum tensile/shear and cross-tension loads of the welds were 3589 and 3419 N, respectively. For a step pin, with increasing rotational speed, the tensile/shear load decreased slightly while the cross-tension load increased.
• The shear fracture and tensile/shear mixed fracture modes were observed under the tensile/shear loading, while the nugget debonding and pull-out failure modes were shown under cross-tension loading.

## Declarations

### Author contribution statement

Tiwan: Conceived and designed the experiments; Performed the experiments.

M. Noer Ilman: Contributed reagents, materials, analysis tools or data.

Kusmono: Analyzed and interpreted the data; Wrote the paper.

### Funding statement

This work was supported by Ministry of Education and Culture, Republic of Indonesia through Doctoral Scholarship to one of us (Mr. Tiwan) with the contract number 2787/UN6.D/LT/2018.

### Data availability statement

Data included in article/supplementary material/referenced in article.

### Declarations of interests statement

The authors declare no conflict of interest.

### Additional information

No additional information is available for this paper.
